# Effect of microbial muramidase supplementation in diets formulated with different fiber profiles for broiler chickens raised under various coccidiosis management programs

**DOI:** 10.1016/j.psj.2023.102955

**Published:** 2023-07-25

**Authors:** Cristiano Bortoluzzi, Estefania Perez-Calvo, Peter B. Olsen, Sharon van der Vaart, Ellen van Eerden, Jerome Schmeisser, Irene Eising, Phokela Segobola, José-Otávio B. Sorbara

**Affiliations:** ⁎ DSM Nutritional Products, Kaiseraugst, Switzerland; † Novozymes A/S, Lyngby, Denmark; ‡ Schothorst Feed Research, Lelystad, the Netherlands; § DSM Nutritional Products, Village-Neuf, France

**Keywords:** broiler, intestine, microbiota, muramidase, peptidoglycans

## Abstract

The objective of the present study was to determine the effects of muramidase (**MUR**) supplemented to diets formulated with different fiber sources (inert or fermentable) on the growth performance and intestinal parameters of broiler chickens raised under different coccidiosis management programs. A total of 2,208 male Ross 308 broilers were housed in 96 floor pens and distributed into a 2 × 3 × 2 factorial arrangement in a completely randomized block design with 2 sources of fiber (inert or fermentable fiber), 3 coccidiosis management programs (none, vaccine, or Salinomycin), and with or without supplementation of MUR at 35,000 LSU(F)/kg of diet. Body weight gain (**BWG**), feed intake (**FI**), and feed conversion ratio (**FCR**) were calculated for each feeding phase (d 0–14, d 14–28, d 28–36) and from d 0 to 36. On d 17 and d 31, samples were taken to analyze several parameters. The experimental data were analyzed with 3-way ANOVA considering the main effect of fiber source, coccidiosis program, inclusion of MUR, and their interactions using JMP 16.2. 16S rDNA sequencing of the ileal and cecal content was carried out to analyze the diversity, composition, and predictive function of the microbiota. From d 0 to 36, BWG increased (*P* = 0.05) by 2.5% in birds supplemented with Salinomycin (*P* = 0.04), and by 2.2% with MUR supplementation (*P* = 0.02). Salinomycin and MUR improved FCR (*P* < 0.0001) when compared to nonsupplemented birds. The supplementation of MUR, regardless of the coccidiosis management program, reduced the intestinal viscosity (*P* = 0.03). On d 31, the highest blood concentration of carotenoids was observed in chickens fed diets supplemented with Salinomycin. MUR led to significant changes in the diversity, composition, and predictive function of the ileal microbiota, mainly on d 31. The results observed herein further explain the positive effects of MUR on the growth performance of broiler chickens.

## INTRODUCTION

The supplementation of precision ingredients and enzymes in the diets of broiler chickens with the objective to promote intestinal health and reduce the negative effects of dietary components on the gastrointestinal tract (**GIT**) functionality of broiler chickens has received attention in the last years (Brugaletta et al., 2022; Dal Pont et al., 2022; Kogut, 2022). The presence of different dietary components, such as mycotoxins, rancid fat, protease inhibitors, phytate, and nonstarch polysaccharides (**NSP**) are known to impact different aspects of the intestinal health (Dal Pont et al., 2022). For example, Kogut et al. (2018) explained that the excess of dietary carbohydrates and fat leads to excess of production of succinate and citrate from the TCA cycle, which may induce chronic inflammation mediated by toll-like receptors (**TLRs**), a process known as metabolic inflammation, and leads to intestinal dysbiosis and loss of intestinal barrier function. In an exhaustive review, Dal Pont et al. (2022) highlighted xylanase, β-glucanase, β-mannanase, protease, and phytase, as the main exogenous enzymes that, by targeting dietary components, may indirectly promote intestinal health.

On the other hand, muramidase (**MUR**), an enzyme that does not target a substrate present in the feed, but a substrate already present in the GIT, has also shown positive effects on nutrient absorption, digestibility of dry matter, ash and fat, and intestinal health of broilers (Sais et al., 2020; Wang et al., 2021; Brugaletta et al., 2022; Goes et al., 2022). It has been demonstrated that MUR, by cleaving β-1,4-glycosidic linkages between N-acetylmuramic acid and N-acetyl glucosamine, efficiently hydrolyzes peptidoglycans (**PGN**) from the cell-wall debris of different gram-positive bacteria found in the GIT of broiler chickens (Frederiksen et al., 2021). This action might be helpful to reduce PGN accumulation in the GIT, thus indirectly improving nutrient utilization (Goodarzi Boroojeni et al., 2019; Sais et al., 2020; Pirgoliev et al., 2021; Goes et al., 2022). Moreover, depolymerization of PGN activates nucleotide-binding oligomerization domain-containing protein 2 (**NOD2**) receptors that play an important role in the maintenance of intestinal homeostasis by downregulating inflammatory responses (Wang et al., 2021). By modulating the intestinal immune system, MUR may indirectly modulate the intestinal microbial community, as shown by Wang et al. (2021) and Brugaletta et al. (2022). Another potential indirect effect of MUR in chickens is the change of cecal and plasma metabolites profile as observed by Brugaletta et al. (2022). These authors observed a decrease in cecal hypoxanthine concentration, followed by its reduction in the blood, which may indicate less oxidative stress, and increase of energy-generating metabolites. These factors may partially explain the consistent improvement in growth performance of broiler chickens promoted by MUR (Goodarzi Boroojeni et al., 2019; Sais et al., 2020; Pirgoliev et al., 2021; Brugaletta et al., 2022; Goes et al., 2022).

Experimental conditions aiming to create mild intestinal challenges are common across studies designed to evaluate feed additives in broiler chickens. A mild challenge can be induced by formulating diets with different profiles or high inclusion of dietary fiber (**DF**), since, by definition, DF is only partially hydrolyzed by the endogenous enzymatic processes of nonruminant animals (González-Alvarado et al., 2007). Dietary fermentable or soluble fiber possesses high water holding capacity which increases intestinal viscosity and tends to bind nutrients from the diet (González-Alvarado et al., 2007), resulting in more substrate available for the intestinal bacteria, increased bacterial turnover, and reduced broiler performance. For example, soy hulls (**SH**) contain up to 13% of soluble fiber (Kim et al., 2015). Insoluble fibers, on the other hand, such as oat hulls (**OH**), are metabolically inert and contain a higher content of lignin than soluble fiber (González-Alvarado et al., 2007), are less detrimental do poultry, and may be, at low concentrations, beneficial to intestinal health.

Coccidiosis vaccination of broiler chickens with live attenuated oocysts initiates an immune response and may induce mild intestinal lesions (Zaheer et al., 2022) leading to a short-term decrease in broiler performance. Ionophores, on the other hand, have efficiently been used as part of coccidiosis management programs due to several advantages associated with their use (Chapman and Rathinam, 2022). Ionophores may also possess antimicrobial activity against gram-positive bacteria (Bafundo et al., 2008), potentially increasing the concentration of PGN in the GIT as a substrate for the action of MUR. It was, therefore, hypothesized that the supplementation of MUR could mitigate the negative effects of diets formulated with fermentable fiber as well as coccidiosis vaccination in broiler chickens, and would beneficially interact with ionophores. The objective of the present study was to determine the effects of MUR supplemented to diets formulated with different fiber sources (inert or fermentable) on the growth performance and intestinal parameters of broiler chickens raised under different coccidiosis management programs (none, vaccine, or ionophore). The parameters analyzed were growth performance, intestinal viscosity, blood carotenoids, ileal lamina propria thickness and Goblet cells number, expression of immune-related genes, and ileal and cecal microbiota.

## MATERIALS AND METHODS

### Animals, Housing, and Experimental Design

The study was conducted at the broiler facilities of Schothorst Feed Research (**SFR**), Lelystad, the Netherlands. The study followed the guidelines of Animal and Human Welfare Codes/Laboratory practice codes in the Netherlands, and the protocol was approved by the Ethics Review Committee (AVD246002016450).

A total of 2,208 male Ross 308 broilers were housed in 96 floor pens covered with wood shavings. The experiment consisted of a 2 × 3 × 2 factorial arrangement in a completely randomized block design with 2 sources of fiber (inert or fermentable fiber), 3 coccidiosis management programs (none, vaccine, or Salinomycin), and with or without supplementation of MUR (Balancius, DSM Nutritional Products, Inc., Kaiseraugst, Switzerland) at 35,000 LSU(F)/kg of diet. Therefore, the experiment comprised of 12 treatments with 8 replicates/treatment, and 23 birds/replicate. At the day of placement, the respective groups were vaccinated against coccidiosis (Paracox 5, MSD) at the recommended dose via spray. The groups under ionophore supplementation were fed diets supplemented with Salinomycin at 70 g/MT.

Pens had an area of 2 m^2^ equipped with a feeder (capacity of 20 kg) and 3 drinking nipples. The birds were inspected at least once a day by an animal caretaker. In case of mortality, pen number and body weight were recorded. The environment temperature was gradually decreased from 34.5°C at placement to 19.4°C at 36 d of age. Room temperature and relative humidity were recorded daily. The light was continuous for the first 24 h. After that, the light schedule was 2D:22L during 1 d, and after 48 h it was changed to 4D:10L:2D:8L during the remaining experimental period, complying with the EU legislation of a minimum of 6 h of darkness with at least one period of 4 h uninterrupted darkness. The birds had *ad libitum* access to a 3-phase wheat-based pelleted diet and drinking water. Temperature and relative humidity were monitored daily. The birds were daily inspected for general health, and deviations from the normal health status were recorded. Birds in poor condition were euthanized for welfare reasons. All the birds in the experiment were vaccinated against Newcastle Disease (Avinew, batch 9AVWK11E) on d 17 via spray, and against Infectious Bursal Disease (Nobilis Gumboro D78 batch A158NJ01) on d 23 via drinking water.

### Feed Manufacturing, Sampling, and Analyses

The diets were manufactured by ABZ Diervoeding, Leusden, the Netherlands, under responsibility of SFR. The composition was based on corn-wheat-soybean meal. The diets were formulated without antimicrobial growth promoters, but, depending on the treatments, contained Salinomycin as a coccidiostat. All the diets included phytase (Ronozyme HiPhos, DSM Nutritional Products, Inc., Kaiseraugst, Switzerland); carbohydrase (Ronozyme WX, DSM Nutritional Products, Inc., Kaiseraugst, Switzerland); and carotenoid (Carophyll yellow 10%, DSM Nutritional Products, Inc., Kaiseraugst, Switzerland). The carotenoid included in the feed was intended to be used as a biomarker of intestinal absorptive capacity and to have its concentration analyzed in the blood. The composition and the calculated nutrients of the starter, grower, and finisher diets are shown in Table 1. A basal mix for both the inert and fermentable fiber sources was made for each feeding phase, and the tested products (MUR, Salinomycin, or both) were added according to each experimental group. When necessary, the tested product was replaced by a filler. The test product or filler was premixed into a 5 kg portion of basal feed and then added to the rest of the feed. After thorough mixing, all feeds were pelleted. Pelleting temperatures were between 65°C and 76°C in the starter diets, between 68°C and 77°C in the grower diets, and between 63°C and 68°C in the finisher diets. In addition, samples were taken for further analysis, as described below.

**Table 1:** Composition and nutritional content of the basal diets.

Ingredients, %		Starter (d 0–14)		Grower (d 14–28)		Finisher (d 28–36)	
		Inert	Fermentable	Inert	Fermentable	Inert	Fermentable
Wheat		40.80	41.10	42.33	43.79	44.06	45.29
Soybean meal		25.64	27.00	27.53	25.85	25.59	23.96
Corn		14.52	14.52	15.00	15.00	15.00	15.00
Oat hulls		8.00	–	8.00	–	8.00	–
Soybean hulls		–	8.00	–	8.00	–	8.00
Corn gluten meal		4.24	2.00	–	–	–	–
Palm oil		–	–	2.07	2.19	2.24	2.39
Soybean oil		2.77	3.43	1.93	1.99	2.25	2.35
Limestone		0.92	0.81	0.63	0.54	0.59	0.61
Monocalcium phosphate		0.61	0.61	0.10	0.11	0.01	0.03
Salt		0.15	0.23	0.21	0.21	0.23	0.26
Sodium bicarbonate		0.21	0.10	0.10	0.10	0.07	0.04
Methionine (99%)		0.19	0.22	0.22	0.23	0.20	0.21
Lysine HCl (79%)		0.28	0.26	0.20	0.25	0.19	0.24
Threonine (98%)		0.05	0.06	0.05	0.08	0.05	0.07
Valine (99%)		0.01	0.03	0.02	0.05	0.02	0.04
Filler^1^/MUR		0.04	0.04	0.04	0.04	0.04	0.04
Filler^2^/Salinomycin		0.58	0.58	0.58	0.58	0.58	0.58
Feed additives premix^3^		0.50	0.50	0.50	0.50	0.50	0.50
Vit and min premix^4^		0.50	0.50	0.50	0.50	0.40	0.40
Total		100.0	100.0	100.0	100.0	100.0	100.0

Nutrient content							

AMEn	kcal/kg	2,850	2,850	2,900	2,900	2,950	2,950
Dry matter	g/kg	882	882	881	881	881	882
Ash	g/kg	49	47	42	39	39	38
Crude protein	g/kg	217	216	202	202	194	194
Crude fiber	g/kg	53	60	64	67	69	72
Crude fiber	g/kg	44	49	44	49	44	48
Starch	g/kg	347	342	354	358	363	367
Sugar	g/kg	31	32	32	32	32	31
NDF	g/kg	147	134	150	136	150	137
ADF	g/kg	64	65	65	65	64	65
Ca	g/kg	7.60	7.60	5.70	5.70	5.32	5.75
P	g/kg	4.76	4.77	3.57	3.56	3.30	3.28
Lys, dig	g/kg	11.00	11.00	10.50	10.50	10.00	10.00
Met+Cis, dig	g/kg	8.03	8.03	7.67	7.67	7.30	7.30
Thr, dig	g/kg	7.15	7.15	6.83	6.83	6.50	6.50
Trp, dig	g/kg	2.17	2.19	2.19	2.12	2.10	2.03

1 The filler was replaced by MUR at the dose level of 35,000 mg LSU(F) per kg diet.

2 The filler was replaced by Salinomycin at the dose level of 70 mg per kg diet.

3 The premix (based on a 0.5% dose): Ronozyme HiPhos (2%); Ronozyme WX (1%); Carophyll yellow (0.12%); corn (96.88%).

4 Vitamin and mineral premix supplied per kg diet: Vitamin A 10,000 IU; vitamin D3 3,333 IU; vitamin E 50 mg; vitamin K3 2.5 mg; vitamin B1 2.5 mg; vitamin B2 7.5 mg; vitamin B6 5 mg; vitamin B12 25 mcg; Niacin 50 mg; D-pantothenic acid 15 mg; choline chloride 500 mg; folic acid 1.5 mg; biotin 0.25 mg; Fe 50 mg (as FeSO_4_·H_2_O); Cu 12.5 mg (as CuSO_4_·5H_2_O); Mn 75 mg (as MnO); Zn 70 mg (as ZnSO_4_·H_2_O); I 2 mg (as Ca(IO_3_)_2_); Se 0.25 mg (as Na_2_SeO_3_); antioxidants (Luctanox EF 5 mg; BHT 2.01 mg, propyl gallate 0.17 mg).

### Measurements—Diets

Analyses of diets were performed by SFR to determine moisture, ash, crude protein, fat, crude fiber, starch, sugar, ADF, NDF, lignin, and in vitro starch degradation. Analyses were performed using different methods, as shown in Table 2, and the results are shown in Table 3.

**Table 2:** Reference methods for nutrient analyses in diets.

Parameter	Determination method	Code
Moisture	Gravimetrically after drying at 80°C vacuum to a constant weight	NEN-ISO 6496:1999
Crude ash	Gravimetrically after ashing the sample for 3 h at 550°C	NEN-ISO 5984:2003
Crude protein (CP)	Determination of the total nitrogen content by combustion according to the Dumas principle and calculation of the crude protein content (CP = N*6.25)	NEN-ISO 16634-2:2016
Crude fat (FATh)	Animal feeding stuffs— Determination of fat content after acid hydrolysis step for fat extraction	NEN-ISO 6492:1999
Crude fiber (CF)	Animal feeding stuffs—Determination of crude fiber content—Method with intermediate filtration	NEN-EN-ISO 6865:2001
Starch	Enzymatic determination of total starch content	NEN-EN-ISO 15914:2005
Sugar	Determination of reducing sugar, crude total sugar, saccharose and lactose	NEN 3571:1974
ADF	Determination of acid detergent fiber (ADF) content	NEN-EN-ISO 13906:2008
NDF	Determination of amylase-treated neutral detergent fiber content (aNDF)	NEN-EN-ISO 16472:2006
Lignin	Determination of acid detergent lignin content	NEN-EN-ISO 13906:2008

**Table 3:** Calculated (Calc.) and analyzed (Ana.) nutrients in the basal diets without coccidiostat and muramidase per fiber source.

		Starter, d 0–14		Grower, d 14–28		Finisher, d 28–36	
		Inert	Fermentable	Inert	Fermentable	Inert	Fermentable
DM^1^, g/kg	Calc.	882	882	881	881	881	882
	Ana.	887	884	903	905	904	907
Ash, g/kg	Calc.	49	47	42	39	39	38
	Ana.	49	49	46	45	42	42
CP^2^, g/kg	Calc.	217	216	202	202	194	194
	Ana.	213	216	200	205	192	201
CF^3^, g/kg	Calc.	53	60	64	67	69	72
	Ana.	50	57	54	57	61	62
CFib^4^, g/kg	Calc.	44	49	44	49	44	48
	Ana.	49	50	43	46	43	50
Starch, g/kg	Calc.	347	342	354	358	363	367
	Ana.	349	347	382	378	384	382
Sugar, g/kg	Calc.	31	32	32	32	32	31
	Ana.	46	46	44	46	44	44
NDF^5^, g/kg	Calc.	147	134	150	136	150	137
	Ana.	131	119	114	112	123	122
ADF^6^, g/kg	Calc.	64	65	65	65	64	65
	Ana.	65	68	55	64	61	69
Lignin, g/kg	Calc.	10	5	10	5	10	5
	Ana.	13	9	15	12	10	10

1 DM: dry matter.

2 CP: crude protein.

3 CF: crude fat.

4 CFib: crude fiber.

5 NDF: neutral detergent fiber.

6 ADF: acid detergent fiber.

### Measurements—Growth Performance

All birds and feed were weighed per pen on d 14, 28, and 36. Body weight gain (**BWG**), feed intake (**FI**), and feed conversion ratio (**FCR**) were calculated per pen for each experimental period (d 0–14, d 14–28, and d 28–36) and considering the cumulative period (d 0–36).

### Sample Collection

Samples of blood from the jugular vein, whole jejunum content, duodenum and ileal tissue were taken from 1 randomly selected bird (at the day of sampling) per pen (except for viscosity) on d 17 and 31, after CO_2_ asphyxiation.

### Jejunal Viscosity

On d 17 and 31, 2 birds and 1 bird, respectively, were randomly selected (at the day of sampling) from each pen and sacrificed to collect the whole jejunum content for viscosity analysis. Viscosity (in centipoise, **cP**) was measured in supernatant (0.5 mL) of jejunum contents (vortexed at 4°C, 3,500 rpm, 10 min) in a cone and plate viscometer (Brookfield Viscometer, Model DV-II+).

### Total Carotenoids

Total blood carotenoid concentration (mg/L) was measured on d 17 and d 31 using the iCheck Carotene device and standard method according to the manufacturer (iCheck, BioAnalyt GmbH, Germany). In short, after CO_2_ asphyxiation, whole blood was collected from the jugular vein into EDTA vials. Thus, 0.4 mL of blood was injected in the special iEx vial, mixed for 10 s, rested for 5 min for the second phase separation and the extractions of the carotenoids from the sample. Finally, total carotenoids content was measured in the iCheck Carotene device.

### Ileal Histology

Ileum samples were collected on d 17 and d 31 and immediately fixed into 4% formaldehyde. Twenty-four hours after sampling, samples were transferred into 50% ethanol and were processed within a week. The samples were embedded in paraffin and blocks were sent to Rennes University-H2P2 Histopathology Platform (Rennes, France) for lamina propria thickness and Goblet cell count analysis. Samples were cut into 4 µM sections. A periodic acid Schiff (**PAS**) stain was used to visualize the Goblet cells, and hematoxylin was used as a counterstain to visualize the villi. After digitalization of the slides, the software NDPI viewer (Hamamatsu) was used for the lamina propria measurements (thickness measured in µm). The Goblet cells were counted using the software HALO (Indica Labs, Albuquerque, NM). The results were expressed as the number of Goblet cells per 100 µm of villi length.

### Immunoglobulins, Alpha-1-Acid Glycoprotein, and Plasma Antioxidant Capacity

Immunoglobulin (**Ig**) A, G, M, and alpha-1-acid glycoprotein (**AGP**) concentrations were analyzed in the serum at d 17 and 31 following manufacturer recommendations using chicken specific ELISA kits (ref. KA2031, KA2426, KA1956, KA2423, respectively; Abnova, Paris, France).

Plasma antioxidant capacity (**PAT**) was also measured with the FRAS 5 (Free Radical Analytic System) instrument, using PAT test (H&D, Parma, Italy). The PAT test measures the iron-reducing power via a colorimetric reaction. Briefly, 40 µL of ferric nitrate was added to a cuvette containing a chromogenic mixture and a first absorbance measure was taken. Ten microliters of plasma was then added to the cuvette and a second absorbance measure was taken after 1 min incubation at 37°C. The test results in U Cor were printed by the instrument (1U Cor = 1.4 µmol/L vitamin C).

### Gene Expression

Duodenum and ileum tissue samples were collected on d 17 and d 31 for gene expression analysis. Total RNA was extracted from tissues (stored at −20°C in RNA later) by lysing tissue with FastPrep 24 (MP Biomedicals, Illkirch, France), using the phenol-chloroform method (TRIzol reagent; Invitrogen, CergyPontoise, France) followed by purification using RNeasy columns by automated method with the Qiacube HT (Qiagen, Courtaboeuf, France). The concentration of RNA was measured by NanoDrop ND-1000 Spectrophotometer (ThermoFisher Scientific, Illkirch, France) and the purity was estimated by A260/A280 ratio. RNA integrity was assessed by using the Agilent 2100 Bioanalyzer (Agilent Technologies, Basel, Switzerland). The threshold of the RNA integrity number (**RIN**) was set at 7.5 to validate sufficient quality of the RNAs. The reference genes used as endogenous control for normalization, gene expression evaluation and gene expression stability in all samples were the glyceraldehyde-3-phosphate dehydrogenase (**GAPDH**) and TATA-Box binding protein (**TBP**). Primer specificity and amplification efficiency were verified for each gene. Data were analyzed using the efficiency-corrected delta-delta-Ct method (Livak and Schmittgen, 2001). The fold-change values of the genes of interest were normalized relative to the values of 2 housekeeping genes. Targeted genes primer list is presented in Table 4 and is based on the QIAGEN Gastro Intestinal Functionality PCR Array.

**Table 4:** Primer sequences used for the real time PCR analysis.

		Gene	Amplicon size
IFN-γ	Forward	CTGGACAGAGAGAAATGAGAAAAGG	99
	Reverse	TTGATGTGCGGCTTTGACTTG	
NoS2	Forward	ACTGAAGGTGGCTATTGGGC	97
	Reverse	GGGTGGGTGGGGTAGTGATA	
IL-12α	Forward	AGGTGCAGAAGCAGAGGACG	87
	Reverse	TTGTGTTGCTCTGACTGTTGGT	
TLR-4	Forward	CACCCTGGACTTGGACCTCA	109
	Reverse	ATGGATGTGGCACCTTGAAAGA	
TNF-α	Forward	TGCTATGAGTCAGGAGCGTTG	75
	Reverse	CTGAAGATCCTAGCATTCCCCCA	
MUC2	Forward	AACCCAGCAGTCAACGACAA	100
	Reverse	GGTTGATACTGTGGAGCCTGA	
GAPDH	Forward	TGAGAAAGTCGGAGTCAACGG	237
	Reverse	GGGTCACGCTCCTGGAAGATA	

### Microbiota Analysis—DNA Extraction, 16S Sequencing, and Bioinformatics

Ileal and cecal microbiota were analyzed in the samples taken on d 17 and d 31. The DNA extraction was carried out with the NucleoSpin 96 soil kit (Macherey-Nagel) following the manufacturer's instructions. Prior to DNA extraction the samples were heat-inactivated at 95°C for 15 min. The generation of OTU tables was done with usearch version 10.0.240 (Edgar, 2010). Primer binding regions were removed with fastx_truncate and reads were filtered to contain less than 1 error per read. The quality filtered reads were denoised with unoise3. OTU abundance was calculated by mapping with usearch_global using a 97% identity threshold. Taxonomical classification was done with the RDP classifier version 2.12. The phylogenetic tree was made by aligning the 16S sequences with mafft version 7.505 and the tree was inferred by FastTree version 2.1.11. For beta- and alpha-diversity analysis, the rarefy_even_depth method from the R phyloseq package was used to rarefy samples to the same depth. The depth for the cecum samples were set to 10,000 reads and the depth for the ileum samples were set to 5,000 reads.

The Shannon diversity was calculated by using the estimate_richness function from the phyloseq package. The effect on treatment on alpha diversity was tested with a generalized linear model as implemented in the R function glm from the stats package. Beta diversity was analyzed by calculating Unifrac distances. Variance analysis of beta diversity was done with Adonis (Permutational Multivariate Analysis) from the vegan package. The detection of differentially abundant bacterial genera was done with a generalized linear model as implemented in the R function glm from the stats package. Predicted functionality of the gut microbiota was assessed using PICRUSt2 generated Kyoto Encyclopedia of Genes and Genomes (**KEGG**) orthologs and pathways (Douglas et al., 2020).

### Statistical Analysis

Data were checked for homogeneity of variances and normality of residues. Individual observations (not the entire experimental unit) were marked as outlier to be excluded from the dataset prior to statistical analyses if the residual (fitted − observed value) was more than 2 × standard error of the parameter. For growth performance parameters and for the additional analyses, pen, and individual birds, respectively, were considered the experimental unit. The experimental data were analyzed with 3-way ANOVA considering the main effect of fiber source, coccidiosis management program, inclusion of MUR, and their interactions using JMP (Version 16.2. SAS Institute Inc., Cary, NC, 1989–2021). In case of an interaction between the factors, LSMeans Tukey procedure was used to separate the means. Values were considered significant with *P* ≤ 0.05, and as a trend with 0.05 < *P* ≤ 0.10.

## RESULTS

### Growth Performance

The growth performance results are shown in Tables 5 and 6. During the starter phase (d 0–14) a 3-way interaction was observed for FCR (*P* = 0.04) wherein the supplementation of MUR to inert fiber diets without coccidiosis management program, the supplementation of Salinomycin to inert fiber diets with and without MUR supplementation, and the supplementation of MUR to fermentable fiber diets under coccidiosis vaccination or Salinomycin supplementation promoted the best FCR (Table 5). The worst FCR was observed for broilers fed inert fiber diets without coccidiosis management program and without MUR supplementation. There was a main effect of coccidiosis management program (*P* = 0.005) and MUR supplementation (*P* = 0.03) on BWG. Salinomycin supplementation promoted higher BWG compared to coccidiosis vaccination, and the birds supplemented with MUR presented 2.6% better BWG compared to nonsupplemented birds. During the grower phase (d 14–28), there was a 3-way interaction on FI (*P* = 0.04) and FCR (*P* = 0.006). Birds under coccidiosis vaccination fed the inert fiber diet had a significantly better FCR when supplemented with MUR than without MUR. In fermentable fiber diets, FCR was significantly better in chickens supplemented with MUR and Salinomycin compared to chickens without coccidiosis management program and without MUR (Table 5).

**Table 5:** Effect of the dietary treatments on the growth performance from 0 to 14 and 14 to 28 d.

Fiber	Coccidiosis program	MUR (LSU/F)/kg	BWG, g	FI, g	FCR	BWG, g	FI, g	FCR
			0–14 d			14–28 d		

Inert	None	0	382	532	1.400^a^	1,079	1,541^b^	1.406abcde
Inert	None	35,000	397	527	1.280^b^	1,172	1,657^a^	1.404bcde
Inert	Vaccine	0	379	490	1.294^ab^	1,125	1,617^ab^	1.439^a^
Inert	Vaccine	35,000	386	493	1.327^ab^	1,185	1,628^ab^	1.375^e^
Inert	Salinomycin	0	394	500	1.268^b^	1,191	1,658^a^	1.378cde
Inert	Salinomycin	35,000	390	496	1.242^b^	1,153	1,594^ab^	1.376^de^
Ferm.	None	0	381	478	1.306^ab^	1,163	1,659^a^	1.427^ab^
Ferm.	None	35,000	395	532	1.347^ab^	1,149	1,622^ab^	1.413abc
Ferm.	Vaccine	0	360	485	1.346^ab^	1,161	1,618^ab^	1.389cde
Ferm.	Vaccine	35,000	389	508	1.272^b^	1,197	1,678^a^	1.403bcde
Ferm.	Salinomycin	0	404	530	1.338^ab^	1,165	1,663^a^	1.409abcde
Ferm.	Salinomycin	35,000	400	498	1.240^b^	1,203	1,677^a^	1.380cde
SEM			8.1	16.6	0.04	28.8	35.3	0.01
Inert			388	506	1.302	1,151	1,616	1.396
Ferm.			388	505	1.308	1,173	1,653	1.403
SEM			3.3	6.8	0.02	11.7	14.4	0.01
	None		389^ab^	517	1.333	1,141	1,620	1.412
	Vaccine		378^b^	494	1.310	1,167	1,635	1.401
	Salinomycin		397^a^	506	1.272	1,178	1,648	1.386
	SEM		4.1	8.3	0.02	14.4	17.7	0.01
		0	383^b^	502	1.325	1,147	1,626	1.408
		35,000	393^a^	509	1.285	1,176	1,643	1.392
		SEM	3.3	6.8	0.02	11.7	14.4	0.01

*P* values								

Fiber*CocciPrg*MUR			0.49	0.17	0.04	0.08	0.04	0.006
Fiber*CocciPrg			0.24	0.20	0.69	0.89	0.92	0.18
Fiber*MUR			0.40	0.37	0.89	0.59	0.83	0.36
CocciPrg*MUR			0.12	0.17	0.76	0.44	0.36	0.59
Fiber			0.95	0.89	0.78	0.18	0.07	0.32
CocciPrg			0.005	0.15	0.10	0.17	0.52	0.01
MUR			0.03	0.47	0.08	0.08	0.40	0.02

a–e Values without a common superscript letter within a column differ significantly (*P* < 0.05). Ferm: fermentable fiber diet. CocciPrg: coccidiosis program; MUR: muramidase; BWG: body weight gain; FI: feed intake; FCR: feed conversion ratio.

**Table 6:** Effect of the dietary treatments on the growth performance from 28 to 36 and 0 to 36 d.

Fiber	Coccidiosis program	MUR LSU(F)/kg	BWG, g	FI, g	FCR	BWG, g	FI, g	FCR
			28–36 d			0–36 d		

Inert	None	0	851	1,400	1.646	2,312	3,473	1.504
Inert	None	35,000	866	1,430	1.658	2,456	3,614	1.471
Inert	Vaccine	0	871	1,439	1.654	2,375	3,546	1.493
Inert	Vaccine	35,000	850	1,411	1.659	2,414	3,540	1.466
Inert	Salinomycin	0	863	1,400	1.623	2,448	3,557	1.445
Inert	Salinomycin	35,000	925	1,439	1.562	2,468	3,530	1.431
Ferm.	None	0	877	1,486	1.702	2,421	3,642	1.504
Ferm.	None	35,000	914	1,487	1.630	2,458	3,642	1.482
Ferm.	Vaccine	0	874	1,463	1.675	2,395	3,565	1.489
Ferm.	Vaccine	35,000	902	1,511	1.679	2,481	3,698	1.491
Ferm.	Salinomycin	0	913	1,471	1.614	2,490	3,618	1.472
Ferm.	Salinomycin	35,000	889	1,450	1.631	2,492	3,626	1.455
SEM			21.8	25.8	0.03	40.5	53.5	0.01
Inert			871	1,420^b^	1.634	2,412^b^	3,543^b^	1.468^b^
Ferm.			895	1,478^a^	1.655	2,456^a^	3,632^a^	1.482^a^
SEM			8.9	10.5	0.01	16.5	21.9	0.001
	None		877	1,451	1.659^a^	2,412^b^	3,593	1.490^a^
	Vaccine		874	1,456	1.667^a^	2,416^b^	3,587	1.485^a^
	Salinomycin		897	1,440	1.607^b^	2,474^a^	3,582	1.451^b^
	SEM		10.9	12.9	0.01	20.2	26.8	0.01
		0	875	1,443	1.653	2,407^b^	3,567	1.484^a^
		35,000	891	1,455	1.637	2,462^a^	3,608	1.466^b^
		SEM	8.9	10.5	0.01	16.5	21.9	0.001

*P* values								

Fiber*CocciPrg*MUR			0.07	0.13	0.07	0.37	0.17	0.40
Fiber*CocciPrg			0.60	0.67	0.91	0.92	0.97	0.19
Fiber*MUR			0.83	0.88	0.94	0.56	0.86	0.20
CocciPrg*MUR			0.74	0.98	0.59	0.34	0.50	0.40
Fiber			0.06	<0.0001	0.13	0.05	0.005	0.004
CocciPrg			0.25	0.66	0.002	0.04	0.96	<0.0001
MUR			0.19	0.42	0.27	0.02	0.18	0.0001

a–b Values without a common superscript letter within a column differ significantly (*P* < 0.05). Ferm: fermentable fiber diet. CocciPrg: coccidiosis program; MUR: muramidase; BWG: body weight gain; FI: feed intake; FCR: feed conversion ratio.

From d 28 to 36 (finisher phase), no interactions between the factors were observed (*P* > 0.05). However, a main effect of fiber source was observed on FI (*P* < 0.0001), wherein the fermentable fiber-based diet increased FI by 58 g. The coccidiosis management program significantly affected FCR (*P* = 0.002), with the best outcome observed when the birds were supplemented with Salinomycin (Table 6). Considering the entire experimental period, d 0 to 36, no interactions were observed for any of the studied variables. However, main effects of fiber source, coccidiosis management program, and MUR supplementation were observed for BWG and FCR, and also a main effect of fiber source on FI (Table 6). BWG increased (*P* = 0.05) by 1.8% in birds fed diets formulated with fermentable fiber, by 2.5% in birds supplemented with Salinomycin when compared to the other coccidiosis management programs (*P* = 0.04), and by 2.2% with MUR supplementation vs. nonsupplemented birds (*P* = 0.02). Diets formulated with fermentable fiber increased FI of the birds by 2.5% (*P* = 0.005) when compared to inert fiber fed birds. Regarding FCR, it was observed that fermentable fiber-based diets led to a worse FCR (*P* = 0.004) vs. inert fiber-based diets. Salinomycin inclusion improved FCR (*P* < 0.0001) by 2.3 and 2.6% when compared to coccidiosis vaccinated birds or without any method of coccidiosis control, respectively. Lastly, MUR supplemented birds showed an improved FCR (*P* = 0.0001) when compared to nonsupplemented birds (Table 6).

### Ileal Morphology, Jejunal Viscosity, and Blood Carotenoids

There were neither interactions nor main effects (*P* > 0.05) on any of the analyzed ileal morphology parameters (lamina propria thickness and Goblet cell numbers; Supplementary Table 1). Regarding the jejunal content viscosity (Table 7), on d 17, an interaction between fiber source by coccidiosis management program was observed (*P* < 0.0001), wherein birds fed inert fiber-based diets presented the lowest viscosity values, regardless of the coccidiosis management program, while within the fermentable fiber fed birds, Salinomycin and coccidiosis vaccination reduced the viscosity by 40 and 15%, respectively, when compared to birds under no coccidiosis management program (Supplementary Table 2). On d 31, an interaction between coccidiosis management program by MUR supplementation was observed on the viscosity of the ileal content (*P* = 0.03). The supplementation of MUR, regardless of the coccidiosis management program, and coccidiosis vaccination without MUR supplementation, reduced the viscosity when compared to Salinomycin fed birds without MUR supplementation (Supplementary Table 2).

**Table 7:** Effect of the dietary treatments on the jejunum viscosity (cP) and total blood carotenoid concentration (mg/L) of broiler chickens at different ages.

Fiber	Coccidiosis program	MUR LSU(F)/kg	Jejunal viscosity, d 17	Jejunal viscosity, d 31	Blood carotenoids, d 17	Blood carotenoids, d 31
Inert	None	0	3.16	2.95	2.15	1.71abcd
Inert	None	35,000	3.09	2.79	2.38	1.56bcd
Inert	Vaccine	0	3.23	3.04	1.84	1.32^cd^
Inert	Vaccine	35,000	2.69	2.90	1.77	2.29^ab^
Inert	Salinomycin	0	3.29	3.28	2.28	2.51^a^
Inert	Salinomycin	35,000	3.14	2.93	2.35	2.29^ab^
Ferm.	None	0	7.84	3.76	1.50	1.05^d^
Ferm.	None	35,000	7.15	3.63	1.26	1.53bcd
Ferm.	Vaccine	0	6.14	3.64	1.22	1.08^d^
Ferm.	Vaccine	35,000	6.56	3.53	1.53	1.35^cd^
Ferm.	Salinomycin	0	5.19	5.20	1.55	1.99abc
Ferm.	Salinomycin	35,000	3.81	3.24	1.91	2.04abc
SEM			0.70	0.36	0.70	0.36
Inert			3.10	2.98	2.13^a^	1.95
Ferm.			6.12	3.83	1.49^b^	1.51
SEM			0.29	0.15	0.11	0.10
	None		5.31	3.28	1.82^ab^	1.46
	Vaccine		4.65	3.28	1.59^b^	1.51
	Salinomycin		3.86	3.66	2.02^a^	2.21
	SEM		0.35	0.36	0.13	0.12
		0	4.81	3.64	1.76	1.61
		35,000	4.41	3.17	1.87	1.84
		SEM	0.29	0.15	0.11	0.10

*P* values						

Fiber*CocciPrg*MUR			0.09	0.10	0.38	0.04
Fiber*CocciPrg			<0.0001	0.52	0.39	0.60
Fiber*MUR			0.48	0.15	0.80	0.76
CocciPrg*MUR			0.39	0.03	0.80	0.03
Fiber			<0.0001	<0.0001	<0.0001	0.0001
CocciPrg			<0.0001	0.14	0.04	<0.0001
MUR			0.06	0.001	0.42	0.03

a–e Values without a common superscript letter within a column differ significantly (*P* < 0.05). Ferm: fermentable fiber diet. CocciPrg: coccidiosis program; MUR: muramidase.

Blood carotenoid concentration was evaluated on d 17 and d 31 and the results are shown in Table 7. No interaction between the factors was observed (*P* > 0.05) on d 17; however, a main effect of fiber source (*P* < 0.0001) was observed. Chickens fed fermentable fiber-based diet showed a lower carotenoid concentration compared to birds fed inert fiber-based diets. In addition, a main effect of coccidiosis management program (*P* = 0.04) was observed, wherein the vaccination against coccidiosis reduced the carotenoid concentration compared to Salinomycin supplemented birds. On d 31, however, a 3-way interaction was observed (*P* = 0.04). The highest blood concentration of carotenoids was observed in chickens fed diets supplemented with Salinomycin in the inert fiber diets without MUR.

### Immunoglobulins, Alpha-1-Acid Glycoprotein, and Plasma Antioxidant Capacity

The results of the serum concentrations of Ig are shown in Table 8. On d 17, an interaction between fiber source and coccidiosis management program was observed on the IgG concentration (*P* = 0.05). The highest IgG concentration was observed in vaccinated birds fed fermentable fiber, and the lowest concentration in vaccinated birds fed inert fiber. There was also an effect of fiber source on the concentration of IgA (*P* = 0.002), wherein chickens fed diets formulated with fermentable fiber presented an increased concentration compared to inert fiber fed birds. Birds that were vaccinated against coccidiosis showed a higher (*P* = 0.005) concentration of serum IgM than birds supplemented with Salinomycin. On d 31, however, the supplementation of Salinomycin, as main effect, reduced the concentration of IgA (*P* = 0.006), IgG (*P* = 0.0005), and IgM (*P* = 0.02) when compared to birds without coccidiosis management program, while chickens vaccinated against coccidiosis showed intermediary results. Additionally, there was an interaction (*P* = 0.02) between fiber source by MUR supplementation on the serum IgG concentration on d 31 (Supplementary Table 3). It was observed that chickens fed inert fiber-based diets and supplemented with MUR showed a higher concentration of IgG than nonsupplemented birds, whereas chickens fed fermentable fiber-based diet presented intermediary values, regardless of MUR supplementation.

**Table 8:** Effect of the dietary treatments on serum concentration of immunoglobulins (Ig) of broiler chickens at different ages.

Fiber	Coccidiosis program	MUR LSU(F)/kg	IgA (µm/mL)	IgG (µm/mL)	IgM (µm/mL)	IgA (µm/mL)	IgG (µm/mL)	IgM (µm/mL)
			17 d			31 d		

Inert	None	0	89	1.38	151	262	2.24	133
Inert	None	35,000	79	1.13	114	242	3.08	172
Inert	Vaccine	0	85	1.03	164	218	2.30	125
Inert	Vaccine	35,000	106	0.93	154	183	2.48	124
Inert	Salinomycin	0	83	1.28	109	173	1.77	136
Inert	Salinomycin	35,000	60	1.50	120	187	2.50	132
Ferm.	None	0	106	1.33	124	218	2.87	153
Ferm.	None	35,000	134	1.25	167	195	2.79	132
Ferm.	Vaccine	0	102	1.32	160	186	2.79	139
Ferm.	Vaccine	35,000	106	1.80	159	178	2.27	114
Ferm.	Salinomycin	0	107	1.28	127	159	1.74	102
Ferm.	Salinomycin	35,000	83	1.17	119	170	1.76	124
	SEM		12.8	0.18	17.5	24.9	0.27	13.9
Inert			84^b^	1.21	135	211	2.40	137
Ferm.			106^a^	1.36	143	184	2.37	127
SEM			5.24	0.07	7.15	10.2	0.11	5.65
	None		102	1.27	139^ab^	229^a^	2.74^a^	147^a^
	Vaccine		100	1.27	159^a^	191^ab^	2.46^a^	125^ab^
	Salinomycin		83	1.31	119^b^	172^b^	1.94^b^	123^b^
	SEM		6.42	0.09	8.75	12.5	0.13	6.92
		0	95	1.27	139	203	2.28	131
		35,000	95	1.29	139	193	2.48	133
		SEM	5.24	0.07	7.15	10.2	0.11	5.65

*P* values								

Fiber*CocciPrg*MUR			0.27	0.35	0.12	0.88	0.95	0.08
Fiber*CocciPrg			0.28	0.05	0.89	0.65	0.28	0.48
Fiber*MUR			0.66	0.59	0.25	0.80	0.02	0.23
CocciPrg*MUR			0.09	0.49	0.92	0.53	0.26	0.44
Fiber			0.002	0.23	0.47	0.07	0.86	0.21
CocciPrg			0.07	0.96	0.005	0.006	0.0005	0.02
MUR			0.96	0.87	0.97	0.48	0.22	0.83

a–b Values without a common superscript letter within a column differ significantly (*P* < 0.05). Ferm: fermentable fiber diet. CocciPrg: coccidiosis program; MUR: muramidase.

There was no 3-way interaction between the factors on the AGP concentration and PAT activity (Supplementary Table 4). There was an effect of coccidiosis management program on the concentration of AGP on d 31 (*P* = 0.01). Chickens raised without coccidiosis management program showed a higher AGP concentration than vaccinated birds (219 vs. 172 µm/mL). Additionally, an interaction of fiber type by coccidiosis management program was observed on PAT on d 31 (*P* = 0.004; Supplementary Table 5), wherein chickens fed inert fiber-based diets and vaccinated against coccidiosis showed higher PAT than birds fed fermentable fiber-based diet under coccidiosis vaccination or supplementation of Salinomycin.

### Gene Expression

The results on the expression of immune-related genes are presented in Supplementary Tables 6 to 9. Regarding duodenum on d 17, there was no interaction between the factors for any gene evaluated (Supplementary Table 6). As main effect, it was observed that feed based on fermentable fiber (*P* = 0.04) and MUR supplementation (*P* = 0.01) upregulated the expression of TLR-4 compared to inert fiber-fed birds and nonsupplemented birds, respectively. Additionally, as a main effect, Salinomycin supplementation downregulated the expression of NoS2 (*P* = 0.04) and TNF-α (*P* = 0.05) compared to birds raised without coccidiosis management program. In the duodenum on d 31 (Supplementary Table 7), a 3-way interaction was observed on the expression of NoS2 (*P* = 0.03). Chickens fed inert fiber-based diets, supplemented with MUR, showed the higher expression when no coccidiosis management program was applied compared to birds supplemented with Salinomycin, or when fed fermentable fiber-based diets, except when supplemented with MUR under no coccidiosis management program or supplemented with Salinomycin. In the ileum on d 17, it was observed that MUR supplementation slightly upregulated the expression of MUC2 (*P* = 0.01; Supplementary Table 8). Lastly, there was a 3-way interaction for the gene expression of NoS2 (*P* = 0.01) and TNF-α (*P* = 0.02) in the ileum on d 31 (Supplementary Table 9). Within the inert fiber-based diet supplemented with MUR, coccidiosis vaccine upregulated the expression of TNF-α, while within the fermentable fiber-based diet without MUR supplementation, coccidiosis vaccination increased its expression compared to birds raised without coccidiosis management program or under Salinomycin supplementation.

### Intestinal Microbiota—Diversity and Composition

#### Ileum—D 17

There was no effect of any of the tested factors on the alpha and beta-diversity indices of the ileal microbiota at d 17 (data not shown).

The complete taxonomic composition of the ileal microbiota at d 17 is shown in Supplementary Table 10. There was no effect of MUR supplementation, coccidiosis vaccination, nor Salinomycin supplementation for any of 100 genera detected. However, fiber source exerted an influence (*P* < 0.05) on 20 of these groups, wherein diets formulated with inert fiber led to a reduction (negative estimate) or increase (positive estimate) of their abundances. Genera that were reduced with inert fiber source include, but are not limited to: *Oceanobacillus, Anaerosalibacter, Novibacillus*, and *Streptococcus*, while *Alistipes, Lachnospiraceae*, and *Flavonifractor* were increased.

#### Cecum—D 17

**Figure 1:**
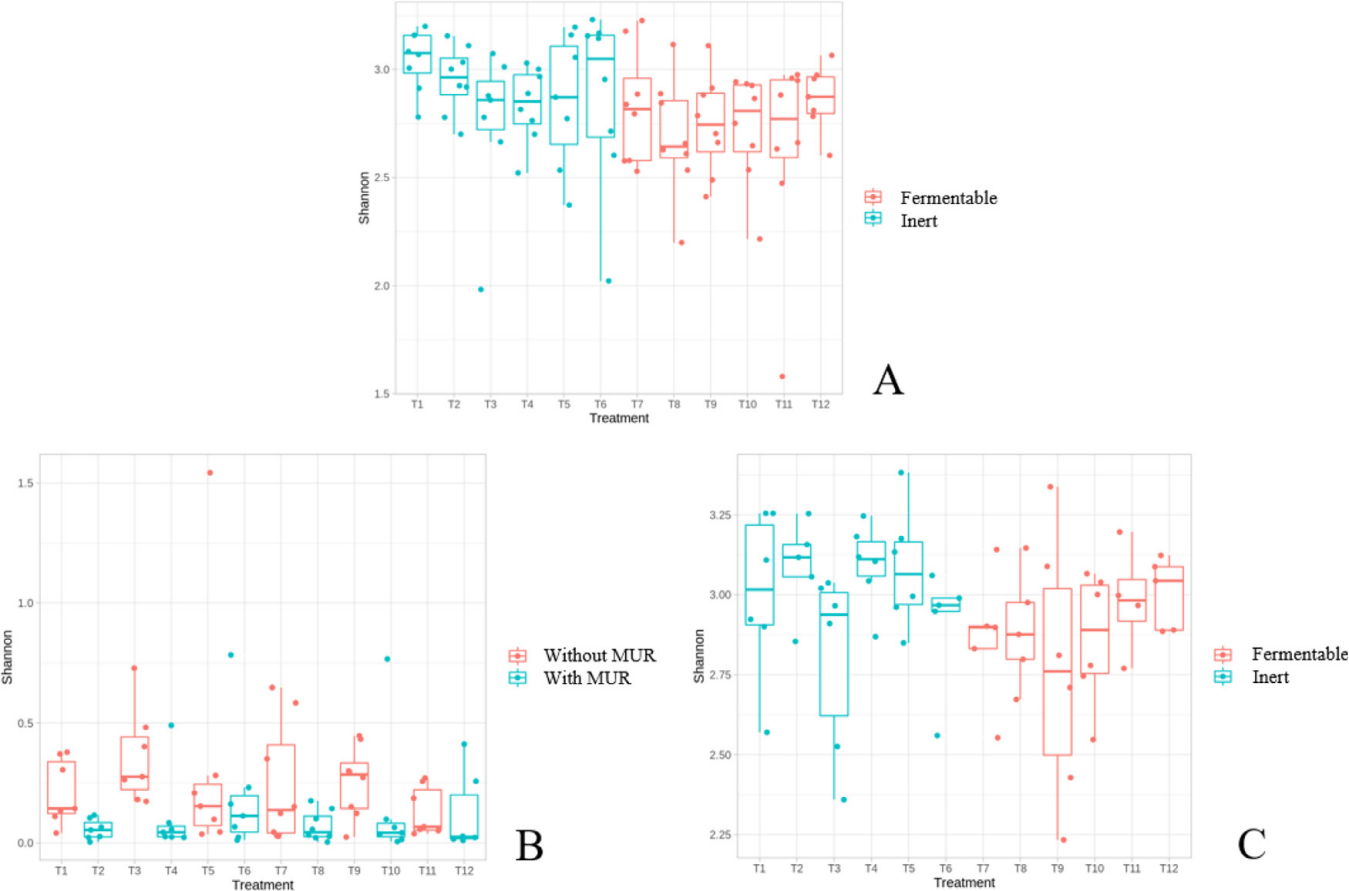
Alpha-diversity index (Shannon index) of the ileal and cecum microbiota of broiler chickens according to the experimental diets. (A) Cecum at 17 d comparing fermentable vs. inert fiber-based diets (*P* = 0.02); (B) Ileum at 31 d comparing the diet with or without supplementation of muramidase (MUR; *P* = 0.005); (C) Cecum at 31 d comparing fermentable vs. inert fiber-based diets (*P* = 0.07). The other comparisons were not significant and, therefore, not shown. Treatments: T1: inert, no coccidiosis program, no MUR; T2: inert, no coccidiosis program, with MUR; T3: inert, coccidiosis vaccine, no MUR; T4: inert, coccidiosis vaccine, with MUR; T5: inert, Salinomycin, no MUR; T6: inert, Salinomycin, with MUR; T7: fermentable, no coccidiosis program, no MUR; T8: fermentable, no coccidiosis program, with MUR; T9: fermentable, coccidiosis vaccine, no MUR; T10: fermentable, coccidiosis vaccine, with MUR; T11: fermentable, Salinomycin, no MUR; T12: fermentable, Salinomycin, with MUR.

**Figure 2:**
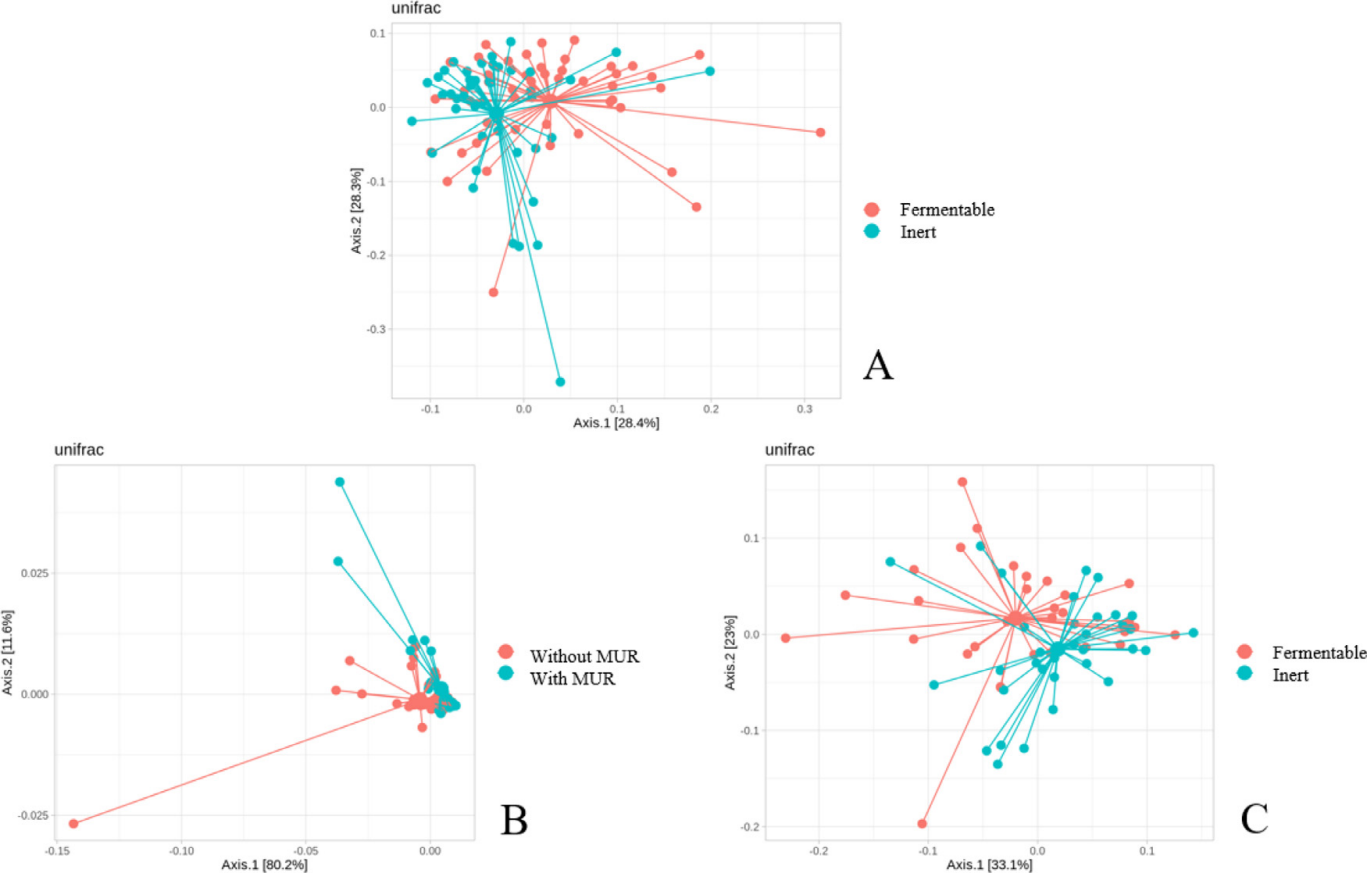
Beta-diversity index of the ileal and cecum microbiota of broiler chickens according to the experimental diets. (A) Cecum at 17 d comparing fermentable vs. inert fiber-based diets (*P* = 0.002); (B) Ileum at 31 d comparing the diet with or without supplementation of muramidase (MUR; *P* = 0.02); (C) Cecum at 31 d comparing fermentable vs. inert fiber-based diets (*P* = 0.02). The other comparisons were not significant and, therefore, not shown.

The alpha-diversity result of the cecal microbiota at d 17 is shown in Figure 1A. It was observed that fermentable fiber-fed broiler showed a lower (*P* = 0.02) alpha diversity (Shannon index) compared to inert fiber-fed broilers. Fiber source also affected the beta diversity (*P* = 0.002) of the cecum at d 17 as shown in Figure 2A.

The complete taxonomic composition of the cecal microbiota at d 17 is shown in Supplementary Table 11. The supplementation of MUR reduced the abundance of the genera *Catenibacillus* (*P* = 0.0002) and tended to reduce *Staphylococcus* (*P* = 0.09; Figure 4A). On the other hand, fiber source exerted an influence (*P* < 0.05) on 8 genera wherein diets formulated with inert fiber led to a reduction (negative estimate) or increase (positive estimate) of their abundances (Supplementary Table 11).

#### Ileum—D 31

The alpha-diversity result of the ileal microbiota at d 31 is shown in Figure 1B. It was observed that the supplementation of MUR reduced (*P* = 0.005) the alpha diversity (Shannon index) compared to nonsupplemented birds. MUR supplementation also affect the beta diversity of the ileal microbiota at d 31 (*P* = 0.02) as shown in Figure 2B.

**Figure 3:**
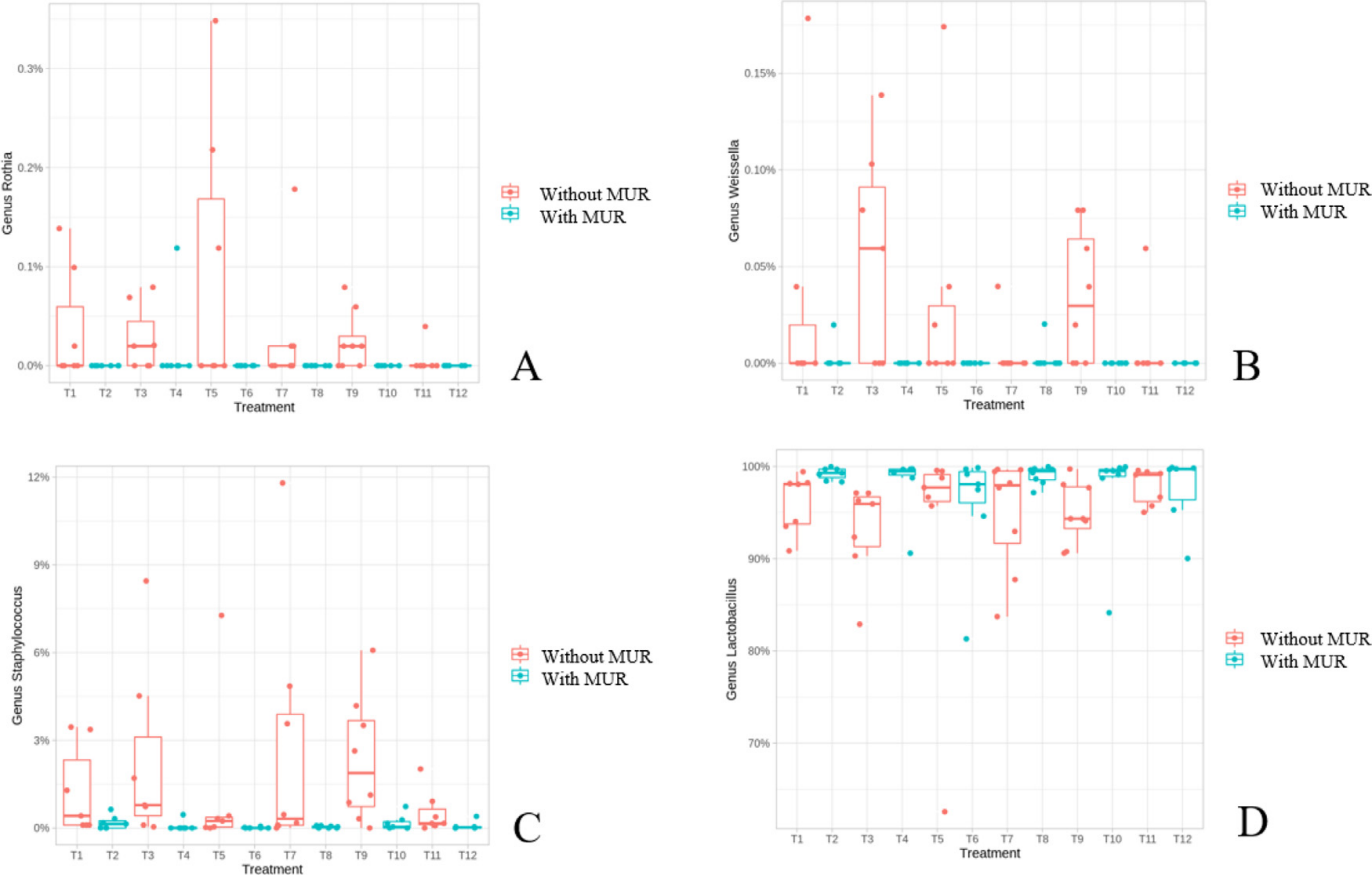
Relative abundance of the genera *Rothia* (A), *Weissella* (B), *Staphylococcus* (C), and *Lactobacillus* (D) in the ileal microbiota at 31 d of broiler chickens supplemented or not with muramidase. Treatments: T1: inert, no coccidiosis program, no MUR; T2: inert, no coccidiosis program, with MUR; T3: inert, coccidiosis vaccine, no MUR; T4: inert, coccidiosis vaccine, with MUR; T5: inert, Salinomycin, no MUR; T6: inert, Salinomycin, with MUR; T7: fermentable, no coccidiosis program, no MUR; T8: fermentable, no coccidiosis program, with MUR; T9: fermentable, coccidiosis vaccine, no MUR; T10: fermentable, coccidiosis vaccine, with MUR; T11: fermentable, Salinomycin, no MUR; T12: fermentable, Salinomycin, with MUR.

The complete taxonomic composition of the ileal microbiota at d 31 is shown in Supplementary Table 12. The supplementation of MUR reduced (negative estimate) or increased (positive estimate) the abundance of 11 genera. The genera *Rothia, Weissella*, and *Staphylococcus* were significantly reduced (*P* < 0.0001), and *Lactobacillus* increased (*P* = 0.007) due to MUR supplementation (Figure 3). Additionally, inert fiber-based diets changed the abundance of 14 genera in total (Supplementary Table 12).

#### Cecum—D 31

The alpha-diversity result of the cecal microbiota at d 31 is shown in Figure 1C. A trend (*P* = 0.07) toward reduced Shannon index in broilers fed fermentable fiber-based diet compared to inert fiber-based diets was observed. However, the type of fiber changed the structure (*P* = 0.02) of the cecal microbiota (beta diversity) as shown in Figure 2C.

**Figure 4:**
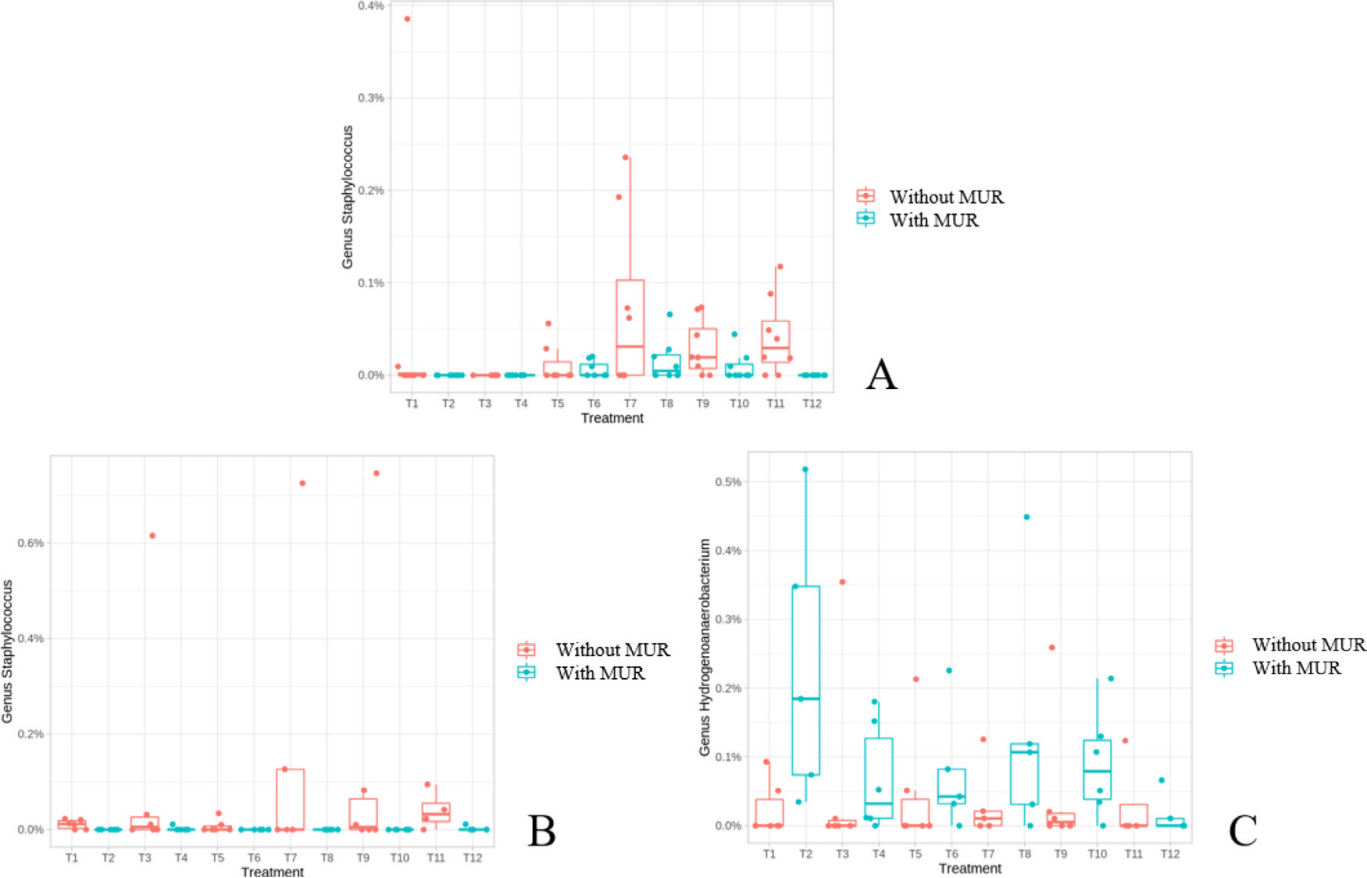
Relative abundance of the genera *Staphylococcus* in the cecum at 17 d (A), and *Staphylococcus* (B), and *Hydrogenoanaerobium* (C) in the cecum microbiota at 31 d of broiler chickens supplemented or not with muramidase. Treatments: T1: inert, no coccidiosis program, no MUR; T2: inert, no coccidiosis program, with MUR; T3: inert, coccidiosis vaccine, no MUR; T4: inert, coccidiosis vaccine, with MUR; T5: inert, Salinomycin, no MUR; T6: inert, Salinomycin, with MUR; T7: fermentable, no coccidiosis program, no MUR; T8: fermentable, no coccidiosis program, with MUR; T9: fermentable, coccidiosis vaccine, no MUR; T10: fermentable, coccidiosis vaccine, with MUR; T11: fermentable, Salinomycin, no MUR; T12: fermentable, Salinomycin, with MUR.

The complete taxonomic composition of the cecal microbiota at d 31 is shown in Supplementary Table 13. The supplementation of MUR reduced (negative estimate) or increased (positive estimate) the abundance of 6 genera. The genera *Staphylococcus* were significantly reduced (*P* = 0.0001), and *Hydrogenoanaerobacterium* increased (*P* = 0.02) with MUR supplementation (Figure 4B and C). Additionally, inert fiber-based diets changed the abundance of 6 genera, and the supplementation of Salinomycin 5 genera (Supplementary Table 13).

### Intestinal Microbiota—Predictive Function

The changes in the predictive functions of the ileal and cecal microbiota are shown in Supplementary Tables 14 to 17. In the ileal microbiota at d 17, the diet formulation with inert fiber changed the abundance of 38 functional pathways (*P* < 0.10; Supplementary Table 14). The top 5 changed pathways were reduced in birds fed inert fiber (negative estimate) and are related to sulfur oxidation, butanediol biosynthesis, heme biosynthesis from glycine, and 4-hydroxyphenylacetate degradation. In the cecum, 6 pathways were changed due to type of fiber, but the supplementation of MUR or Salinomycin did not change any pathways in the ileal or cecal microbiota at d 17 (Supplementary Tables 14 and 15).

On d 31, however, the supplementation of MUR led to a change (*P* < 0.10) of 145 functional pathways in the ileal microbiota (Supplementary Table 16), wherein the top 5 showed negative estimate and are related to formaldehyde assimilation, amino butanoate degradation, formaldehyde oxidation, urea cycle, and butanediol biosynthesis. One pathway of particular interest is related to the TCA cycle (acetate-producers) that was reduced due to MUR supplementation (*P* = 0.00005). Inert fiber-based diet changed the abundance of 19 pathways in the ileal microbiota at d 31. In the cecal microbiota, however, none of factors changed any of the functional pathways (Supplementary Table 17).

## DISCUSSION

Previous work has shown that MUR supplementation in broiler diets improves FCR (Sais et al., 2020; Pirgozliev et al., 2021; Goes et al., 2022), increases nutrient digestibility and absorption (Goodarzi Boroojeni et al., 2019; Sais et al., 2020; Pirgozliev et al., 2021; Goes et al., 2022), downregulates inflammatory responses (Wang et al., 2021) and modulates the intestinal microbial community (Sais et al., 2020; Burgaletta et al., 2022); however, the exact mechanism of this enzyme remains to be fully elucidated. Therefore, this study was designed to evaluate the effects of MUR supplementation on several parameters related to intestinal health in diets formulated with different types of fiber (fermentable vs. inert fiber) and in broilers under varying coccidiosis management programs (vaccine and ionophore) in order to better understand in what conditions MUR, among fiber type and coccidiosis program, works more efficiently.

The present study showed that diets formulated with fermentable fiber promoted a worse FCR than diets formulated with inert fiber, and increased digesta viscosity on both d 17 and 31, but Salinomycin mainly, supplemented to fermentable fiber-fed birds, reduced the viscosity, especially on d 17, most likely as a secondary effect on the GIT. A similar pattern was observed by Tejeda and Kim (2021a) wherein the inclusion of fermentable fiber (soy hulls) in the diet of chickens increased the intestinal viscosity when compared to inert fiber (cellulose). In addition, Gonzalez-Alvarado et al. (2007) showed that the inclusion of soy hulls increased the jejunum content viscosity when compared to oat hull-based diets. The inclusion of fermentable type of fiber to the diets of broilers may have negative effects on the performance and intestinal health by reducing the passage rate and increasing the availability of nutrients to microorganisms, and potentially, pathogenic bacteria (Tejeda and Kim, 2021b). Supplementation of Salinomycin also provided better BWG and FCR compared to the coccidiosis vaccinated birds. The response of the coccidiosis vaccine was not unexpected since it is known that coccidiosis vaccine may lead to worse performance of broilers when compared to Salinomycin (Orso et al., 2022), due to the lesions caused by the coccidia during its cycling. Orso et al. (2022) showed a reduction of 147 g in coccidiosis vaccinated birds vs. Salinomycin, and 4.5 points worse FCR. Moreover, fermentable fiber-based diet and coccidiosis vaccine reduced total carotenoid concentration in the blood, highlighting that these factors interfere with nutrient absorption and consequently are detrimental to growth performance of the birds.

MUR supplementation improved the BWG by 55 g in the current study, besides the enhancement in FCR, confirming the results observed in previous studies (Goodarzi Boroojeni et al., 2019; Sais et al., 2020; Pirgozliev et al., 2021; Goes et al., 2022) where this effect was mainly explained due to a better nutrient digestibility and absorption. Although digestibility was not measured in the current experiment, blood carotenoid concentration, used as biomarker related to absorption capacity and intestinal health (Rochell et al., 2016), and intestinal viscosity were evaluated. Results showed an increase in carotenoid concentration, especially at d 31 (14%), with MUR supplementation. In addition, within inert fiber-based diets, MUR increased carotenoid concentration of vaccinated birds, and Salinomycin supplementation increased its concentration, regardless of MUR inclusion. Within fermentable fiber-based diets, only Salinomycin increased carotenoid concentration, regardless of MUR inclusion. Similarly, Goes et al. (2022) showed an increase in total blood carotenoids of broilers supplemented with MUR. These authors also reported a lower passage of FITC-d from the intestinal lumen to the blood in the MUR supplemented group of broilers, indicating lower intestinal permeability.

On the other hand, despite the fact that fermentable fiber increased intestinal viscosity, and likely bacterial turnover associated to an increase of PGN in the intestinal lumen, no interaction between fiber source and MUR supplementation was observed. This can be probably explained by the fact that other feed enzymes, such as carbohydrases which reduce intestinal viscosity, were included in the basal diets. However, an interaction showed that MUR reduced the viscosity of the digesta on d 31, regardless of the coccidiosis management program, and overall, MUR supplementation reduced the intestinal viscosity by 13%. Previous work demonstrated that MUR reduced intestinal viscosity of broiler chickens and this effect was even more evident with the combination with xylanase (Pérez-Calvo et al., 2019).

Goes et al. (2022) demonstrated that the supplementation of broilers with MUR improved the growth performance of the birds, partially due to improvement in the digestibility of dry matter, fat, and ash. It is known that MUR, despite being a feed enzyme, does not have a direct effect on the nutrients of the diet, but instead, may act mitigating the intestinal inflammation, by degrading bacterial PGN that produces muramyl-dipeptide (**MDP**; Wang et al., 2021). These authors demonstrated that MUR-treated PGN originated from *Staphylococcus aureus* was able to activate NOD2 receptors in HEK293 cells, which was not observed with cells treated only with PGN or MUR alone. Acute stimulation of NOD2 receptors by MDP may lead to a proinflammatory response; however, chronic exposure of immune cells to MDP, as normally occurs in the intestine of broilers, may mediate a tolerance state to bacterial products (Hedl et al., 2007). The GIT of broiler chickens is an environment with chronic exposure to bacterial products that are in constant process of turnover. The increased degradation of PGN by MUR may promote an overstimulation of NOD2 receptors that ultimately lead to a state of tolerance. In the present study, and in the presence of both stressors, fermentable fiber and coccidiosis vaccination, MUR downregulated the expression of TNF-α, a proinflammatory cytokine.

Feed formulation with fermentable fiber that increases intestinal viscosity, and likely bacterial turnover, may be a factor that increases PGN availability in the intestinal lumen. Nevertheless, Salinomycin, that also has effect against gram-positive bacteria (Bafundo et al., 2008), could augment the concentration of PGN to serve as a substrate for MUR, and lead to modulation of inflammation. In the current study, diets formulated with fermentable fiber led to an increase in serum concentration of IgA, the main immunoglobulin in the intestinal mucosa, that suggests greater immune stimulation. Dal Pont et al. (2023) hypothesized that earlier than 14 d of age, high fermentable type of fiber drives an innate response in chickens, shifting to a more adaptative response afterward, with reduction of macrophage stimulatory cytokines, and increase in chronic inflammation-related cytokines, such as IL-16 and IL-10. This observation is justifiable by the fact at d 17, IgA was already elevated in the serum of fermentable fiber-fed chickens. Therefore, such evaluations at earlier ages are essential to show how MUR works during the acute inflammatory response promoted by dietary components.

Despite being able to degrade PGN, MUR does not seem to cause bacterial cell lysis (Wang et al., 2021), but changes in the diversity and composition of the ileal and cecal microbiota were observed (Wang et al., 2020; Brugaletta et al., 2022), most likely due to an indirect mechanism. Our results partially agree with Sais et al. (2020) and Brugaletta et al. (2022), wherein MUR supplementation reduced the alpha diversity as measured by the Shannon index in the cecal content microbiota. In our study, MUR reduced the Shannon index in the ileal microbiota only, with no effect on the cecal microbiota. However, diet formulation with fermentable fiber, reduced the alpha diversity of the cecal microbiota. Similar result was also found on the beta diversity for both fiber and MUR factors. There are many aspects that alter the intestinal microbiota structure (Kers et al., 2018), diet being one of the main factors: either directly by providing nutrients that serve as a substrate for the microorganisms, or indirectly, in the case of feed enzymes that may generate molecules that will be used by the microorganisms or that modulates the immune system. In the present study, MUR reduced the microbiota diversity. Supplementing broiler chicken diets with MUR could, indeed, have an important impact on the microbiota since endogenous lysozyme is one of the most important protective factors in epithelial secretions (Dave et al., 2016).

It is evident that MUR supplementation affected the ileal microbiota, both in terms of diversity and composition, to a larger extent compared to the cecal microbiota which was more affected by the fiber type. The results presented herein showed a strong effect of MUR in reducing several genera such as *Rothia, Weissella*, and *Staphylococcus*, and increasing *Lactobacillus* in the ileal microbiota on d 31. The reduction of *Staphylococcus* by MUR was also observed in the cecal microbiota on d 17 and 31. The genus *Rothia* has been associated with atrophy and intestinal metaplasia (Sung et al., 2020). The reduction of *Weissella*, a lactic acid bacterium that can be linked to improved intestinal health (Cupi and Elvig-Jørgensen, 2019), may have happened in consequence of the increase in *Lactobacillus*, another lactic acid bacterium, highly prevalent in the small intestine, and positively associated with intestinal health. The increase in *Lactobacillus* has also been observed by Lichtenberg et al. (2017) in the cecal microbiota of chickens.

The species within the genus *Staphylococcus* are facultative anaerobes. The abundance of another facultative anaerobe, genus *Jeotgalicoccus*, was also decreased in the ileum at d 31. Similarly, the aerobe genera *Rothia, Corynebacterium*, and *Bacillus* decreased in abundance upon treatment with MUR. Therefore, it is possible to speculate that MUR shifts ileum oxygen concentrations by decreasing aerobes and facultative anaerobe bacteria. To further investigate these observations, PICRUSt2 (Douglas et al., 2020) was applied to predict the functional pathways changes in the microbiota, as described in the methods section. In the ileal microbiota, on d 31, the 2 versions of the TCA cycle pathways, PWY-7254-TCA cycle VII and REDCITCYC-TCA cycle VIII, were significantly decreased in MUR-treated broilers. The possible decrease in oxygen could be a sign of reduced amount of reactive oxygen species (**ROS**) in the ileum. A high concentration of ROS in the intestine of broilers is considered as detrimental for the intestinal health (Mishra and Jha, 2019). In humans, oxygen has been proposed to play a role in inflammatory bowel diseases (Rigottier-Gois, 2013). In line with this hypothesis, bacterial formate oxidation and aerobic respiration pathways were found to be over-represented in a chemically induced murine model of colitis (Hughes et al., 2017). Therefore, these observations may also help to explain the positive effects of MUR on the growth performance of broilers.

## CONCLUSIONS

In conclusion, the results presented herein further explain the role of dietary fiber and coccidiosis vaccination and how they may impair the growth performance of broiler chickens. Fermentable fiber reduced the growth performance of the animals, increased intestinal viscosity, and reduced the absorption of carotenoids. Salinomycin performed better than coccidiosis vaccination in terms of growth performance as well as different parameters of intestinal health. MUR supplementation did not positively interact with Salinomycin nor mitigated the effects of fermentable fiber or coccidiosis vaccination, but in general, improved the growth performance of the chickens and improved the intestinal quality as shown by reduced intestinal viscosity and increased blood concentration of carotenoids. MUR modulated the diversity, composition, and predictive function of the ileal microbiota, especially in older birds, which can also help to explain the mechanism of action of this enzyme in the GIT of broiler chickens.

## Appendix Supplementary materials

Image, application 1

Image, application 2

Image, application 3

## Footnotes

Supplementary materials Supplementary material associated with this article can be found in the online version at doi:10.1016/j.psj.2023.102955.
