# Supplementary material for: Effect of microbial muramidase supplementation in diets formulated with different fiber profiles for broiler chickens raised under various coccidiosis management programs
Source: Poult Sci. 2023 Jul 25;102(10):102955. doi: 10.1016/j.psj.2023.102955 (PMC10440566; doi:10.1016/j.psj.2023.102955)
Supplement: Supplementary file 3 [file mmc3.docx]

Supplementary Table 1. Effect of the dietary treatments on the lamina propria thickness (LPT; µm) and goblet cell count (GCC; n/100 µm villi) in the ileum of broiler chickens at different ages.

| Fiber | CocciPrg | MUR | LPT | GCC | LPT | GCC |
| --- | --- | --- | --- | --- | --- | --- |
|  |  |  | 17 days | | 31 days | |
| Inert | None | 0 | 25.7 | 12.2 | 22.8 | 11.8 |
| Inert | None | 35,000 | 25.5 | 11.3 | 22.9 | 11.5 |
| Inert | Vaccine | 0 | 24.3 | 13.4 | 24.4 | 11.6 |
| Inert | Vaccine | 35,000 | 22.6 | 14.3 | 24.1 | 13.0 |
| Inert | Salinomycin | 0 | 27.7 | 11.3 | 25.5 | 11.2 |
| Inert | Salinomycin | 35,000 | 23.8 | 11.4 | 23.2 | 12.7 |
| Ferm. | None | 0 | 23.5 | 12.8 | 24.9 | 11.7 |
| Ferm. | None | 35,000 | 25.5 | 12.5 | 21.6 | 12.5 |
| Ferm. | Vaccine | 0 | 25.9 | 12.0 | 21.7 | 12.8 |
| Ferm. | Vaccine | 35,000 | 25.0 | 11.6 | 21.3 | 11.8 |
| Ferm. | Salinomycin | 0 | 23.3 | 10.4 | 23.1 | 11.4 |
| Ferm. | Salinomycin | 35,000 | 23.9 | 13.9 | 25.1 | 12.1 |
|  | *SEM* |  | 1.65 | 1.00 | 1.21 | 0.63 |
| Inert |  |  | 24.9 | 12.3 | 23.8 | 12.0 |
| Ferm. |  |  | 24.5 | 12.2 | 23.0 | 12.1 |
| *SEM* |  |  | 0.67 | 0.41 | 0.49 | 0.26 |
|  | None |  | 25.0 | 12.2 | 23.0 | 11.9 |
|  | Vaccine |  | 24.5 | 12.8 | 22.9 | 12.3 |
|  | Salinomycin |  | 24.7 | 11.7 | 24.2 | 11.9 |
|  | *SEM* |  | 0.82 | 0.50 | 0.60 | 0.32 |
|  |  | 0 | 25.0 | 12.0 | 23.7 | 11.7 |
|  |  | 35,000 | 24.4 | 12.5 | 23.0 | 12.3 |
|  |  | *SEM* | 0.67 | 0.41 | 0.49 | 0.26 |
| *P Values* | | | | | | |
| Fiber*CocciPrg*MUR | |  | 0.72 | 0.24 | 0.07 | 0.18 |
| Fiber*CocciPrg | |  | 0.21 | 0.07 | 0.18 | 0.72 |
| Fiber*MUR | |  | 0.21 | 0.43 | 0.87 | 0.35 |
| CocciPrg*MUR | |  | 0.52 | 0.23 | 0.66 | 0.57 |
|  |  |  |  |  |  |  |
| Fiber | |  | 0.67 | 0.82 | 0.26 | 0.80 |
| CocciPrg | |  | 0.89 | 0.30 | 0.24 | 0.56 |
| MUR | |  | 0.48 | 0.41 | 0.33 | 0.17 |

^a-b^ Values without a common superscript letter within a column differ significantly (P<0.05). Ferm: fermentable fiber diet. CocciPrg: Coccidiosis Program; MUR: Muramidase.

Supplementary Table 2. Interactions on the effects of the dietary treatments on ileal viscosity (cP) of broiler chickens at different ages.

| Interaction | |  | Interaction | |  |
| --- | --- | --- | --- | --- | --- |
| Fiber | CocciPrg | Viscosity, d 17 | Coccidiosis program | MUR | Viscosity, d 31 |
| Inert | None | 3.12 ^d^ | None | 0 | 3.35 ^ab^ |
| Inert | Vaccine | 2.95 ^d^ | Vaccine | 0 | 3.34 ^b^ |
| Inert | Salinomycin | 3.21 ^d^ | Salinomycin | 0 | 4.24 ^a^ |
| Ferm. | None | 7.49 ^a^ | None | 35,000 | 3.21 ^b^ |
| Ferm. | Vaccine | 6.35 ^b^ | Vaccine | 35,000 | 3.22 ^b^ |
| Ferm. | Salinomycin | 4.50 ^c^ | Salinomycin | 35,000 | 3.08 ^b^ |
| *SEM* | | 0.50 | *SEM* | | 0.25 |
| Fiber*CocciPrg | | <0.0001 | CocciPrg*MUR | | 0.03 |

^a-d^ Values without a common superscript letter within a column differ significantly (P<0.05). Ferm: fermentable fiber diet. CocciPrg: Coccidiosis Program; MUR: Muramidase.

Supplementary Table 3. Interaction between fiber source and muramidase supplementation on the serum Immunoglobulin G (IgG) concentration at day 31.

| Fiber | MUR | IgG (µm/mL) |
| --- | --- | --- |
| Inert | 0 | 2.10 ^b^ |
| Inert | 35,000 | 2.69 ^a^ |
| Ferm. | 0 | 2.46 ^ab^ |
| Ferm. | 35,000 | 2.27 ^ab^ |
|  | *SEM* | 0.16 |
|  | *P Value* | 0.02 |

^a-b^ Values without a common superscript letter within a column differ significantly (P<0.05). Ferm: fermentable fiber diet. MUR: Muramidase.

Supplementary Table 4. Effect of the dietary treatments on Alpha-1-Acid Glycoprotein (AGP), and plasma antioxidant capacity (PAT) in the serum of broiler chickens at different ages.

| Fiber | CocciPrg | MUR | AGP (µm/mL) | PAT (µm/mL) | AGP (µm/mL) | PAT (µm/mL) |
| --- | --- | --- | --- | --- | --- | --- |
|  |  |  | 17 days | | 31 days | |
| Inert | None | 0 | 86 | 2,615 | 206 | 2,570 |
| Inert | None | 35,000 | 97 | 2,718 | 230 | 2,675 |
| Inert | Vaccine | 0 | 100 | 2,806 | 142 | 2,800 |
| Inert | Vaccine | 35,000 | 88 | 2,597 | 199 | 2,906 |
| Inert | Salinomycin | 0 | 84 | 2,709 | 173 | 2,788 |
| Inert | Salinomycin | 35,000 | 87 | 2,900 | 173 | 2,743 |
| Ferm. | None | 0 | 102 | 2,841 | 208 | 2,730 |
| Ferm. | None | 35,000 | 103 | 2,709 | 230 | 2,988 |
| Ferm. | Vaccine | 0 | 111 | 2,894 | 154 | 2,498 |
| Ferm. | Vaccine | 35,000 | 88 | 2,655 | 194 | 2,540 |
| Ferm. | Salinomycin | 0 | 96 | 2,860 | 201 | 2,634 |
| Ferm. | Salinomycin | 35,000 | 79 | 2,762 | 176 | 2,462 |
|  | *SEM* |  | 12.3 | 111.5 | 23.1 | 124.2 |
|  |  |  |  |  |  |  |
| Inert |  |  | 90 | 2,724 | 187 | 2,747 |
| Ferm. |  |  | 96 | 2,787 | 194 | 2,642 |
| *SEM* |  |  | 5.02 | 45.5 | 9.42 | 50.7 |
|  | None |  | 97 | 2,721 | 219 ^a^ | 2,741 |
|  | Vaccine |  | 97 | 2,738 | 172 ^b^ | 2,686 |
|  | Salinomycin |  | 87 | 2,808 | 181 ^ab^ | 2,656 |
|  | *SEM* |  | 6.15 | 55.7 | 11.5 | 62.1 |
|  |  | 0 | 97 | 2,788 | 181 | 2,670 |
|  |  | 35,000 | 90 | 2,723 | 200 | 2,719 |
|  |  | *SEM* | 5.02 | 45.5 | 9.42 | 50.7 |
| *P Values* | | | | | | |
| Fiber*CocciPrg*MUR | |  | 0.96 | 0.70 | 0.93 | 0.70 |
| Fiber*CocciPrg | |  | 0.88 | 0.81 | 0.89 | 0.004 |
| Fiber*MUR | |  | 0.35 | 0.16 | 0.58 | 0.93 |
| CocciPrg*MUR | |  | 0.42 | 0.22 | 0.21 | 0.25 |
|  |  |  |  |  |  |  |
| Fiber | |  | 0.41 | 0.34 | 0.63 | 0.14 |
| CocciPrg | |  | 0.43 | 0.52 | 0.01 | 0.62 |
| MUR | |  | 0.40 | 0.33 | 0.15 | 0.49 |

^a-b^ Values without a common superscript letter within a column differ significantly (P<0.05). Ferm: fermentable fiber diet. CocciPrg: Coccidiosis Program; MUR: Muramidase.

Supplementary Table 5. Interaction on the effect of the dietary treatments on plasma antioxidant capacity (PAT) in the serum of broiler chickens at day 31.

| Interaction | |  |
| --- | --- | --- |
| Fiber | CocciPrg | PAT, d 31 |
| Inert | None | 2,623 ^ab^ |
| Inert | Vaccine | 2,853 ^a^ |
| Inert | Salinomycin | 2,754 ^ab^ |
| Ferm. | None | 2,859 ^a^ |
| Ferm. | Vaccine | 2,519 ^b^ |
| Ferm. | Salinomycin | 2,548 ^b^ |
| *SEM* | | 56.4 |
| Fiber*CocciPrg | | 0.004 |

^a-b^ Values without a common superscript letter within a column differ significantly (P<0.05). Ferm: fermentable fiber diet. CocciPrg: Coccidiosis Program; MUR: Muramidase.

Supplementary Table 6. Effect of the dietary treatments on the expression of immune-relate genes in the duodenum of broiler chickens at day 17.

| Fiber | CocciPrg | MUR | MUC2 | NoS2 | TLR-4 | TNF-α | IFN-γ | IL-12α |
| --- | --- | --- | --- | --- | --- | --- | --- | --- |
| Inert | None | 0 | 1.00 | 1.00 | 1.00 | 1.00 | 1.00 | 1.00 |
| Inert | None | 35,000 | 1.07 | 1.00 | 1.12 | 0.58 | 0.76 | 0.93 |
| Inert | Vaccine | 0 | 0.98 | 0.95 | 0.96 | 0.65 | 0.91 | 0.72 |
| Inert | Vaccine | 35,000 | 1.02 | 0.83 | 1.02 | 0.93 | 0.68 | 0.76 |
| Inert | Salinomycin | 0 | 0.96 | 0.82 | 0.84 | 0.47 | 1.31 | 0.93 |
| Inert | Salinomycin | 35,000 | 1.11 | 0.76 | 0.81 | 0.65 | 0.87 | 0.80 |
| Ferm. | None | 0 | 1.11 | 0.88 | 0.78 | 0.89 | 1.24 | 0.79 |
| Ferm. | None | 35,000 | 0.98 | 1.36 | 1.39 | 1.67 | 1.48 | 0.90 |
| Ferm. | Vaccine | 0 | 0.98 | 1.01 | 0.98 | 0.77 | 1.51 | 0.80 |
| Ferm. | Vaccine | 35,000 | 1.08 | 0.88 | 1.20 | 1.19 | 0.77 | 0.96 |
| Ferm. | Salinomycin | 0 | 1.11 | 0.77 | 1.07 | 0.60 | 0.66 | 0.75 |
| Ferm. | Salinomycin | 35,000 | 1.20 | 0.79 | 1.27 | 0.50 | 0.99 | 0.86 |
|  | *SEM* |  | 0.08 | 0.15 | 0.14 | 0.29 | 0.24 | 0.12 |
| Inert |  |  | 1.02 | 0.89 | 0.96 ^b^ | 0.71 | 0.92 | 0.86 |
| Ferm. |  |  | 1.08 | 0.95 | 1.12 ^a^ | 0.94 | 1.11 | 0.84 |
| *SEM* |  |  | 0.03 | 0.06 | 0.06 | 0.12 | 0.10 | 0.05 |
|  | None |  | 1.04 | 1.06 ^a^ | 1.07 | 1.04 ^a^ | 1.12 | 0.91 |
|  | Vaccine |  | 1.01 | 0.92 ^ab^ | 1.04 | 0.89 ^ab^ | 0.97 | 0.81 |
|  | Salinomycin |  | 1.09 | 0.78 ^b^ | 1.00 | 0.55 ^b^ | 0.96 | 0.83 |
|  | *SEM* |  | 0.04 | 0.08 | 0.07 | 0.14 | 0.12 | 0.06 |
|  |  | 0 | 1.02 | 0.91 | 0.94 ^b^ | 0.73 | 1.10 | 0.83 |
|  |  | 35,000 | 1.07 | 0.94 | 1.14 ^a^ | 0.92 | 0.93 | 0.87 |
|  |  | *SEM* | 0.03 | 0.06 | 0.06 | 0.12 | 0.10 | 0.05 |
| *P Value* | | | | | | | | |
| Fiber*CocciPrg*MUR | |  | 0.54 | 0.48 | 0.65 | 0.17 | 0.14 | 0.93 |
| Fiber*CocciPrg | |  | 0.62 | 0.83 | 0.22 | 0.46 | 0.07 | 0.26 |
| Fiber*MUR | |  | 0.45 | 0.32 | 0.06 | 0.29 | 0.38 | 0.20 |
| CocciPrg*MUR | |  | 0.44 | 0.22 | 0.32 | 0.74 | 0.28 | 0.79 |
|  | |  |  |  |  |  |  |  |
| Fiber | |  | 0.25 | 0.54 | 0.04 | 0.18 | 0.18 | 0.87 |
| CocciPrg | |  | 0.37 | 0.04 | 0.73 | 0.05 | 0.55 | 0.51 |
| MUR | |  | 0.29 | 0.73 | 0.01 | 0.24 | 0.19 | 0.60 |

^a-c^ Values without a common superscript letter within a column differ significantly (P<0.05). Ferm: fermentable fiber diet. CocciPrg: Coccidiosis Program; MUR: Muramidase.

Supplementary Table 7. Effect of the dietary treatments on the expression of immune-relate genes in the duodenum of broiler chickens at day 31.

| Fiber | CocciPrg | MUR | MUC2 | NoS2 | TLR-4 | TNF-α | IFN-γ | IL-12α |
| --- | --- | --- | --- | --- | --- | --- | --- | --- |
| Inert | None | 0 | 1.00 | 1.00 ^ab^ | 1.00 | 1.00 | 1.00 | 0.83 |
| Inert | None | 35,000 | 1.05 | 1.17 ^a^ | 1.01 | 0.99 | 0.98 | 1.11 |
| Inert | Vaccine | 0 | 0.88 | 0.62 ^ab^ | 0.91 | 0.78 | 0.79 | 0.83 |
| Inert | Vaccine | 35,000 | 1.03 | 0.67 ^ab^ | 0.97 | 1.26 | 0.57 | 0.79 |
| Inert | Salinomycin | 0 | 1.27 | 0.84 ^ab^ | 0.98 | 0.74 | 0.68 | 0.99 |
| Inert | Salinomycin | 35,000 | 1.07 | 0.40 ^b^ | 0.95 | 0.81 | 0.39 | 0.86 |
| Ferm. | None | 0 | 1.10 | 0.49 ^b^ | 0.76 | 0.38 | 0.67 | 1.02 |
| Ferm. | None | 35,000 | 1.13 | 0.63 ^ab^ | 1.00 | 0.92 | 0.59 | 0.75 |
| Ferm. | Vaccine | 0 | 1.20 | 0.45 ^b^ | 0.82 | 1.71 | 0.40 | 0.87 |
| Ferm. | Vaccine | 35,000 | 0.98 | 0.42 ^b^ | 0.88 | 0.63 | 0.44 | 0.99 |
| Ferm. | Salinomycin | 0 | 1.39 | 0.41 ^b^ | 0.89 | 0.18 | 0.51 | 1.04 |
| Ferm. | Salinomycin | 35,000 | 1.20 | 0.90 ^ab^ | 1.10 | 1.10 | 0.59 | 0.74 |
|  | *SEM* |  | 0.13 | 0.16 | 0.09 | 0.40 | 0.13 | 0.13 |
| Inert |  |  | 1.05 | 0.78 | 0.97 | 0.93 | 0.73 | 0.90 |
| Ferm. |  |  | 1.17 | 0.55 | 0.91 | 0.82 | 0.53 | 0.90 |
| *SEM* |  |  | 0.05 | 0.06 | 0.04 | 0.16 | 0.05 | 0.05 |
|  | None |  | 1.07 | 0.82 | 0.94 | 0.82 | 0.81 ^a^ | 0.93 |
|  | Vaccine |  | 1.02 | 0.54 | 0.90 | 1.09 | 0.55 ^b^ | 0.87 |
|  | Salinomycin |  | 1.23 | 0.64 | 0.98 | 0.71 | 0.54 ^b^ | 0.91 |
|  | *SEM* |  | 0.06 | 0.07 | 0.04 | 0.19 | 0.06 | 0.07 |
|  |  | 0 | 1.14 | 0.63 | 0.89 | 0.80 | 0.67 ^a^ | 0.93 |
|  |  | 35,000 | 1.08 | 0.70 | 0.99 | 0.95 | 0.59 ^b^ | 0.87 |
|  |  | *SEM* | 0.05 | 0.06 | 0.04 | 0.16 | 0.05 | 0.05 |
| *P Value* | | | | | | | | |
| Fiber*CocciPrg*MUR | |  | 0.56 | 0.03 | 0.51 | 0.08 | 0.42 | 0.21 |
| Fiber*CocciPrg | |  | 0.97 | 0.03 | 0.45 | 0.68 | 0.07 | 0.55 |
| Fiber*MUR | |  | 0.41 | 0.11 | 0.12 | 0.91 | 0.16 | 0.25 |
| CocciPrg*MUR | |  | 0.43 | 0.75 | 0.89 | 0.38 | 0.95 | 0.38 |
|  | |  |  |  |  |  |  |  |
| Fiber | |  | 0.13 | 0.007 | 0.22 | 0.64 | 0.004 | 0.99 |
| CocciPrg | |  | 0.08 | 0.02 | 0.4 | 0.4 | 0.002 | 0.85 |
| MUR | |  | 0.39 | 0.45 | 0.06 | 0.51 | 0.22 | 0.47 |

^a-b^ Values without a common superscript letter within a column differ significantly (P<0.05). Ferm: fermentable fiber diet. CocciPrg: Coccidiosis Program; MUR: Muramidase.

Supplementary Table 8. Effect of the dietary treatments on the expression of immune-relate genes in the ileum of broiler chickens at day 17.

| Fiber | CocciPrg | MUR | MUC2 | NoS2 | TLR-4 | TNF-α | IFN-γ | IL-12α |
| --- | --- | --- | --- | --- | --- | --- | --- | --- |
| Inert | None | 0 | 1.00 | 1.00 | 1.00 | 1.00 | 1.00 | 1.00 |
| Inert | None | 35,000 | 1.02 | 1.05 | 1.05 | 0.50 | 0.86 | 0.92 |
| Inert | Vaccine | 0 | 1.03 | 1.11 | 0.99 | 0.66 | 0.96 | 1.00 |
| Inert | Vaccine | 35,000 | 1.11 | 0.98 | 0.78 | 1.08 | 0.85 | 0.90 |
| Inert | Salinomycin | 0 | 1.03 | 1.01 | 0.80 | 0.55 | 1.04 | 1.33 |
| Inert | Salinomycin | 35,000 | 1.08 | 1.08 | 0.82 | 0.74 | 0.89 | 1.13 |
| Ferm. | None | 0 | 1.01 | 1.07 | 1.00 | 0.75 | 0.96 | 0.89 |
| Ferm. | None | 35,000 | 1.01 | 1.00 | 0.97 | 1.32 | 1.01 | 0.93 |
| Ferm. | Vaccine | 0 | 1.01 | 1.18 | 1.05 | 0.77 | 0.80 | 0.94 |
| Ferm. | Vaccine | 35,000 | 1.10 | 0.78 | 0.87 | 1.42 | 1.03 | 1.26 |
| Ferm. | Salinomycin | 0 | 0.96 | 0.83 | 0.95 | 0.62 | 0.98 | 1.28 |
| Ferm. | Salinomycin | 35,000 | 1.06 | 0.97 | 0.96 | 0.57 | 0.65 | 0.81 |
|  | *SEM* |  | 0.04 | 0.15 | 0.08 | 0.28 | 0.13 | 0.12 |
| Inert |  |  | 1.04 | 1.04 | 0.91 | 0.75 | 0.93 | 1.05 |
| Ferm. |  |  | 1.02 | 0.97 | 0.97 | 0.91 | 0.90 | 1.02 |
| *SEM* |  |  | 0.02 | 0.06 | 0.03 | 0.12 | 0.06 | 0.05 |
|  | None |  | 1.01 | 1.03 | 1.01 | 0.89 | 0.96 | 0.93 |
|  | Vaccine |  | 1.06 | 1.01 | 0.92 | 0.98 | 0.91 | 1.02 |
|  | Salinomycin |  | 1.03 | 0.97 | 0.89 | 0.62 | 0.89 | 1.14 |
|  | *SEM* |  | 0.02 | 0.08 | 0.04 | 0.14 | 0.07 | 0.06 |
|  |  | 0 | 1.01 ^b^ | 1.03 | 0.97 | 0.72 | 0.96 | 1.07 |
|  |  | 35,000 | 1.06 ^a^ | 0.98 | 0.91 | 0.94 | 0.88 | 0.99 |
|  |  | *SEM* | 0.02 | 0.06 | 0.03 | 0.12 | 0.06 | 0.05 |
| *P Value* | | | | | | | | |
| Fiber*CocciPrg*MUR | |  | 0.87 | 0.75 | 0.89 | 0.27 | 0.39 | 0.13 |
| Fiber*CocciPrg | |  | 0.75 | 0.79 | 0.24 | 0.67 | 0.56 | 0.14 |
| Fiber*MUR | |  | 0.79 | 0.58 | 0.82 | 0.29 | 0.48 | 0.53 |
| CocciPrg*MUR | |  | 0.34 | 0.26 | 0.08 | 0.39 | 0.31 | 0.03 |
|  | |  |  |  |  |  |  |  |
| Fiber | |  | 0.39 | 0.47 | 0.15 | 0.35 | 0.74 | 0.68 |
| CocciPrg | |  | 0.16 | 0.87 | 0.07 | 0.18 | 0.78 | 0.05 |
| MUR | |  | 0.01 | 0.57 | 0.17 | 0.21 | 0.33 | 0.23 |

^a-b^ Values without a common superscript letter within a column differ significantly (P<0.05). Ferm: fermentable fiber diet. CocciPrg: Coccidiosis Program; MUR: Muramidase.

Supplementary Table 9. Effect of the dietary treatments on the expression of immune-relate genes in the ileum of broiler chickens at day 31.

| Fiber | CocciPrg | MUR | MUC2 | NoS2 | TLR-4 | TNF-α | IFN-γ | IL-12α |
| --- | --- | --- | --- | --- | --- | --- | --- | --- |
| Inert | None | 0 | 1.00 | 1.00 ^ab^ | 1.00 | 1.00 ^abc^ | 1.00 | 1.00 |
| Inert | None | 35,000 | 1.14 | 1.12 ^ab^ | 0.94 | 0.76 ^bc^ | 1.04 | 1.37 |
| Inert | Vaccine | 0 | 1.03 | 0.96 ^ab^ | 0.88 | 1.12 ^abc^ | 0.77 | 0.75 |
| Inert | Vaccine | 35,000 | 1.15 | 0.88 ^ab^ | 0.86 | 2.27 ^a^ | 0.67 | 1.66 |
| Inert | Salinomycin | 0 | 1.12 | 0.77 ^b^ | 0.82 | 0.96 ^abc^ | 0.78 | 0.78 |
| Inert | Salinomycin | 35,000 | 0.96 | 1.20 ^ab^ | 0.91 | 0.81 ^bc^ | 1.19 | 0.86 |
| Ferm. | None | 0 | 1.12 | 0.87 ^ab^ | 0.82 | 0.48 ^c^ | 0.59 | 0.80 |
| Ferm. | None | 35,000 | 1.12 | 1.34 ^a^ | 0.94 | 1.20 ^abc^ | 1.27 | 1.27 |
| Ferm. | Vaccine | 0 | 1.23 | 0.88 ^ab^ | 0.79 | 2.09 ^ab^ | 0.85 | 0.86 |
| Ferm. | Vaccine | 35,000 | 1.19 | 1.00 ^ab^ | 0.83 | 0.67 ^bc^ | 0.97 | 0.81 |
| Ferm. | Salinomycin | 0 | 1.11 | 1.01 ^ab^ | 0.91 | 0.18 ^c^ | 0.89 | 0.89 |
| Ferm. | Salinomycin | 35,000 | 0.96 | 0.85 ^ab^ | 1.00 | 1.11 ^abc^ | 0.76 | 0.91 |
|  | *SEM* |  | 0.06 | 0.12 | 0.09 | 0.52 | 0.21 | 0.30 |
| Inert |  |  | 1.06 | 0.99 | 0.90 | 1.15 | 0.91 | 1.07 |
| Ferm. |  |  | 1.12 | 0.99 | 0.88 | 0.95 | 0.89 | 0.92 |
| *SEM* |  |  | 0.03 | 0.05 | 0.04 | 0.21 | 0.09 | 0.12 |
|  | None |  | 1.09 ^ab^ | 1.08 | 0.93 | 0.86 | 0.97 | 1.11 |
|  | Vaccine |  | 1.15 ^a^ | 0.93 | 0.84 | 1.54 | 0.82 | 1.02 |
|  | Salinomycin |  | 1.03 ^b^ | 0.96 | 0.91 | 0.76 | 0.90 | 0.86 |
|  | *SEM* |  | 0.03 | 0.06 | 0.04 | 0.26 | 0.10 | 0.15 |
|  |  | 0 | 1.10 | 0.91 | 0.87 | 0.97 | 0.81 | 0.85 |
|  |  | 35,000 | 1.08 | 1.07 | 0.91 | 1.13 | 0.98 | 1.15 |
|  |  | *SEM* | 0.03 | 0.05 | 0.04 | 0.21 | 0.09 | 0.12 |
| *P Value* | | | | | | | | |
| Fiber*CocciPrg*MUR | |  | 0.54 | 0.01 | 0.78 | 0.02 | 0.14 | 0.41 |
| Fiber*CocciPrg | |  | 0.32 | 0.83 | 0.33 | 0.92 | 0.48 | 0.58 |
| Fiber*MUR | |  | 0.18 | 0.89 | 0.44 | 0.76 | 0.67 | 0.37 |
| CocciPrg*MUR | |  | 0.02 | 0.25 | 0.81 | 0.76 | 0.49 | 0.61 |
|  | |  |  |  |  |  |  |  |
| Fiber | |  | 0.11 | 0.94 | 0.69 | 0.49 | 0.87 | 0.41 |
| CocciPrg | |  | 0.04 | 0.15 | 0.33 | 0.07 | 0.57 | 0.49 |
| MUR | |  | 0.65 | 0.03 | 0.44 | 0.58 | 0.17 | 0.09 |

^a-c^ Values without a common superscript letter within a column differ significantly (P<0.05). Ferm: fermentable fiber diet. CocciPrg: Coccidiosis Program; MUR: Muramidase.
